# Steroid, ascorbic acid, and thiamine in adults with sepsis and septic shock: a systematic review and component network meta-analysis

**DOI:** 10.1038/s41598-021-95386-9

**Published:** 2021-08-04

**Authors:** Ka Man Fong, Shek Yin Au, George Wing Yiu Ng

**Affiliations:** Department of Intensive Care, Queen Elizabeth Hospital, 30 Gascoigne Road, Kowloon, Hong Kong SAR China

**Keywords:** Infectious diseases, Cardiovascular diseases

## Abstract

To assess the effect from individual component in combinations of steroid, ascorbic acid, and thiamine on outcomes in adults with sepsis and septic shock with component network meta-analysis (NMA). We searched PubMed, EMBASE, and the Cochrane Library Central Register of Controlled Trials from 1980 to March 2021 for randomized controlled trials (RCT) that studied the use of glucocorticoid, fludrocortisone, ascorbic acid, and thiamine in patients with sepsis and septic shock. Citations screening, study selection, data extraction, and risk of bias assessment were independently performed by two authors. The primary outcome was short-term mortality. Secondary outcomes were longer-term mortality, time to resolution of shock and duration of mechanical ventilation. Thirty-three RCTs including 9898 patients presented on short-term mortality. In additive component NMA, patients on ascorbic acid alone (RR 0.74, 95% CI 0.57–0.97) or the combination of glucocorticoid and fludrocortisone (RR 0.89, 95% CI 0.80–0.99) had lower short-term mortality, but only the latter was associated with improved long-term mortality (RR 0.89, 95% CI 0.82–0.98). The use of glucocorticoid or the combination of glucocorticoid, ascorbic acid and thiamine hastened resolution of shock. Component NMA showed glucocorticoid (MD − 0.96, 95% CI − 1.61 to − 0.30) but not ascorbic acid or thiamine shortened the time to resolution of shock. Glucocorticoid shortened the duration of mechanical ventilation (MD − 1.48, 95% CI − 2.43 to − 0.52). In adults with sepsis and septic shock, the combination of glucocorticoid and fludrocortisone improved short-term and longer-term mortality. Glucocorticoid shortened the time to resolution of shock and duration of mechanical ventilation. There was no strong evidence supporting the routine use of thiamine and ascorbic acid, but they were associated with minimal adverse effects.

## Introduction

Multiple studies have shown the value of steroid in sepsis and septic shock in terms of hastening the shock reversal^1–4^. Since the publication of the before-after study by Marikl et al., the addition of ascorbic acid and thiamine to hydrocortisone has drawn much attention in the field^5^. Subsequent randomized controlled trials on the use of this triple therapy yielded conflicting results. Furthermore, there was only one study comparing hydrocortisone alone with the triple therapy^6^. Therefore the optimal treatment agent or combinations in patients with sepsis and septic shock remains unclear.

Network meta-analysis (NMA) calculates the combination of direct and indirect estimates of effects and allows comparison of multiple intervention with improved precision. The model was further improved by Welton et al. who proposed the component network meta-analysis (CNMA) in 2009 for multicomponent interventions^7^. CNMA considers separate effect for each of the different component in an intervention^8^. As a relatively new methodology, CNMA has started to gain its acceptance in the medical community but largely limited in the field of psychiatry^7,9–11^. The aim of this systematic review and NMA/CNMA was to evaluate the use of steroids (including glucocorticoid and fludrocortisone), ascorbic acid, and thiamine in adult patients with sepsis and septic shock.

## Methods

We adhered to the *Preferred Reporting Items for Systematic Reviews and Meta-analyses* extension statement for reporting network meta-analyses (PRIMSA-NMA) (Supplementary material Table 1). ^12^ The protocol for this review was registered in the International Prospective Register of Systematic Reviews (CRD42020216665).

### Data sources and searches

We searched PubMed, EMBASE, and the Cochrane Library Central Register of Controlled Trials from 1980 to March 2021 for potentially relevant studies published in English. Our PubMed search strategy is presented in Supplementary Material Table 2. Reference lists of the relevant articles, conference proceedings, and systematic reviews were also reviewed. We included randomized controlled trials (RCT) of adult patients with sepsis and septic shock investigating the use of glucocorticoid (including hydrocortisone, dexamethasone, and methylprednisolone), fludrocortisone, ascorbic acid and thiamine. Sepsis and septic shock were defined by the individual authors in the included studies. We excluded studies comparing the methods of administration (e.g. bolus vs. infusion) of steroid. We excluded studies enrolling healthy volunteers or animals and studies which excluded patients with septic shock.

### Study selection and data extraction

Two authors (KF and SA) independently screened citations and abstracts in duplicate and independently. All references judged potentially relevant were evaluated for full-text eligibility. Discrepancies were solved by consensus with the third author (GN). When relevant data or information were missing, we attempted to contact the authors of the studies.

### Outcome measures

The primary outcome was the short-term mortality (< 90 days). The secondary outcomes were longer term mortality (≥ 90 days), time to resolution of shock and duration of mechanical ventilation. Tertiary outcomes included the ICU length of stay, hospital length of stay and adverse events (including secondary infections, gastrointestinal bleeding, delirium, hyperglycemia, hypernatremia, and any potential complications related to ascorbic acid and thiamine).

### Risk of bias assessment

Two authors (SA and GN) independently assessed the risk of bias of included studies. We assessed the risk of bias of the RCT using the revised Cochrane risk-of-bias tool for randomized trials^13^. In case of disagreement for the attribution of risk of bias, it was solved by discussion and consensus with the third author (KF).

### Statistical analysis and quality of evidence

We performed a random effect NMA and additive CNMA using a frequentist framework. Additive CNMA allows each component to have it separate effect thus the total effect of an intervention is equal to the sum of individual component effect^9^. We calculated the mean differences (MD) for continuous outcomes and risk ratios (RR) for dichotomous outcomes. Where data were not available, we converted the median and interquartile range to mean and standard deviations using a published equation^14^. We also ranked the treatment using the P-score which was based on the frequentist point estimates and their standard errors^15^. We assessed the assumption of transitivity by comparing the distribution effect modifier across studies. Potential inconsistency in the random effect model was assessed by node-splitting method design-by-treatment model^16^. Publication bias was investigated using the Egger’s test and comparison-adjusted funnel plots. We performed sensitivity analysis by limiting to 3 categories: (1) studies with low risk of bias, (2) studies recruiting > 50% of patients dependent on inotrope/ vasopressor (because of the difference in the proportion of patients with septic shock across studies), (3) studies recruiting patients after 2016 (because of the major practice change after the publication of the 2016 Surviving Sepsis Campaign)^17^. Lastly, (3) we also performed sensitivity analysis by excluding studies using high dose glucocorticoid (≥ 400 mg/day hydrocortisone or equivalent, according to previous literature^18^). Since the dose of glucocorticoid were weight-based in some studies^1,2,19–23^, the dose would be calculated based on a 60 kg man in the analysis. The NMA was performed using the package ‘netmeta’ (version 1.3–0) in R (version 4.0.3, The R Foundation for Statistical Computing).

We applied the modified *Grading of Recommendations Assessment, Development and Evaluation* (GRADE) approach for network meta-analysis^24,25^. The contributions of all direct estimates to the network estimates were evaluated from the contribution matrix^26^. We rated down the quality of evidence when intransitivity was present, or when there was incoherence between direct and indirect estimates. When both direct and indirect evidence were available, we chose the higher of the two quality ratings for the NMA estimate^24^.

## Results

### Literature search

The initial search yielded 2563 citations; 69 proved potentially eligible after reviewing the full-text articles. Thirty-four studies met our inclusion criteria, representing 9,992 patients (Supplemental Information Fig. 1). The characteristics of the included studies are shown in Supplemental Information Table 3. Trial sample size ranged from 27 to 3713. Results of the individual studies can be found in Supplemental Information Table 4 to Table 14.

**Figure 1:**
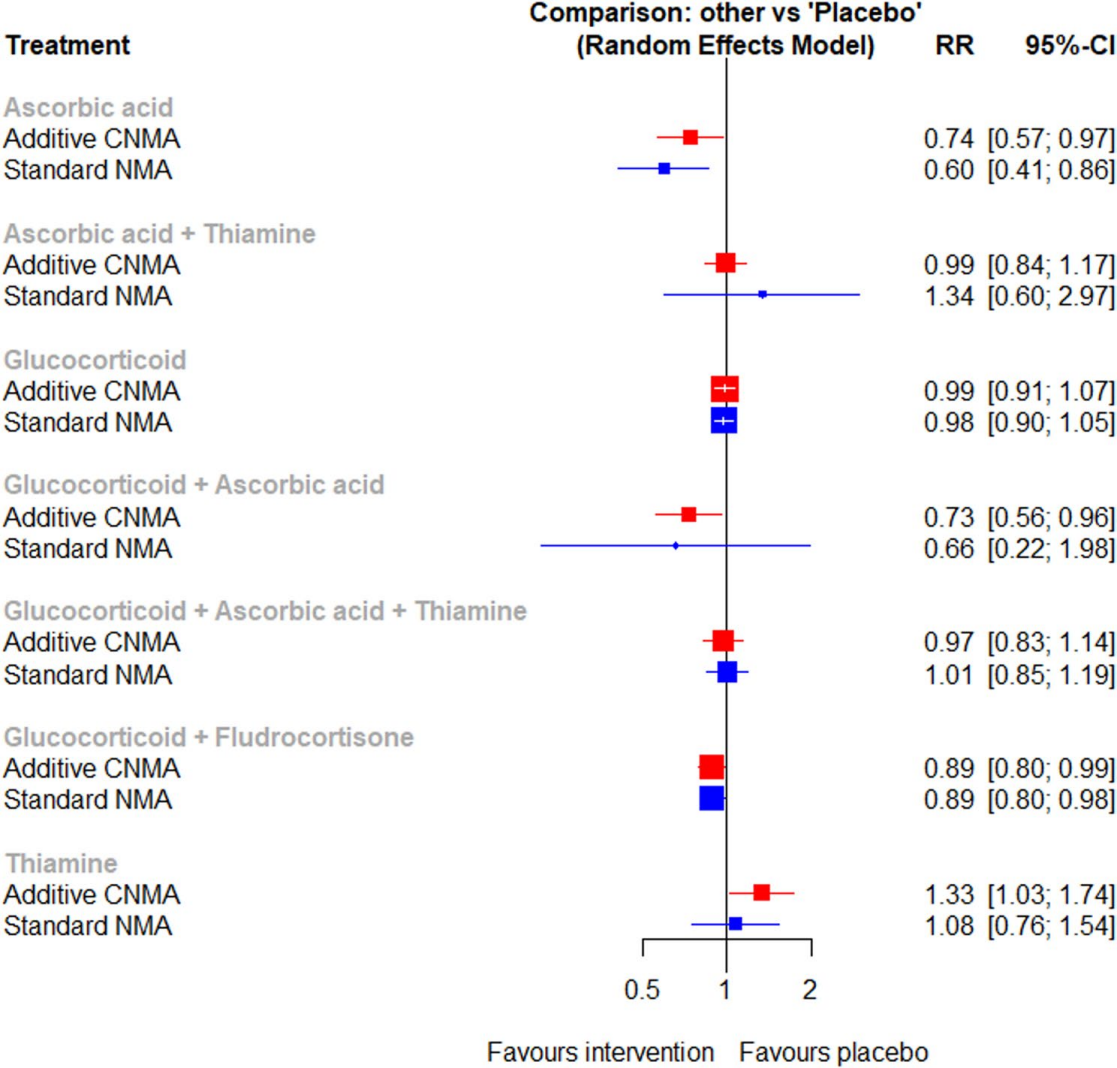
Network meta-analysis on short-term mortality (< 90 days).

### Risk of bias and quality of evidence

There were 12 studies adjudicated at low risk of bias in all domains. The summary of risk of bias assessment is shown in Supplemental Information Fig. 2.

**Figure 2:**
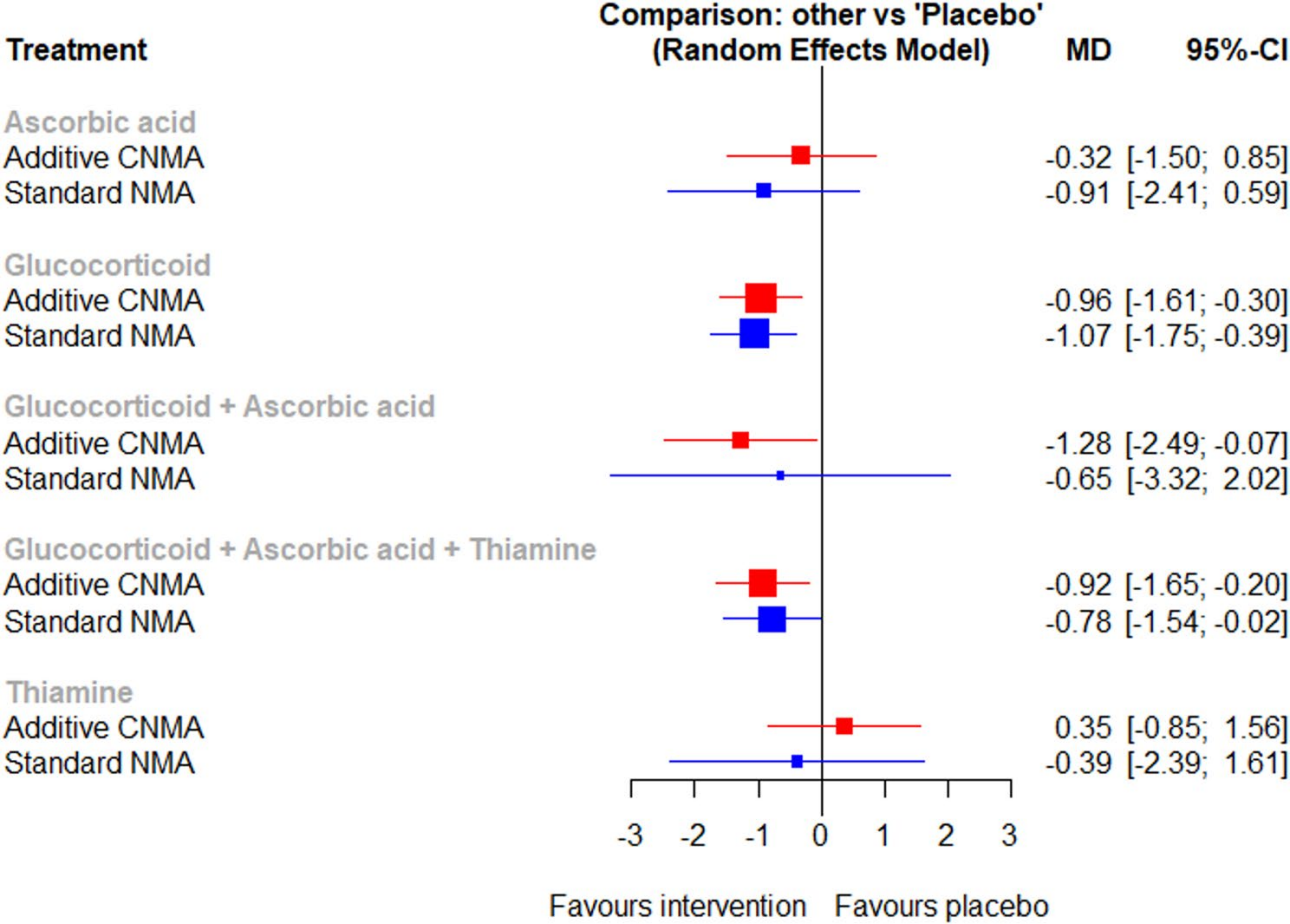
Network meta-analysis on time to resolution of shock.

### Assessment of transitivity and inconsistency

Supplemental Information Table 15 shows the important trial and patient characteristics (Dosage of glucocorticoid, age, APACHE II, percentage of vasopressor/ inotrope dependence at baseline) across treatment comparisons for assessment of transitivity. On visual inspection the dosage of glucocorticoid, age, and APACHE II appeared similarly distributed across comparisons, although there was missing information. However, 2 out of 34 studies recruited less than 50% of patients dependent on vasopressors/ inotropes at baseline. Otherwise, the assumption of transitivity appeared to be valid. There was no significant incoherence detected by statistical testing nor visual inspection of direct and indirect estimates (Supplemental Information Table 16).

### Primary outcome

Thirty-three studies representing 9,898 patients reported the short-term mortality (< 90 days)^1–4,6,19–23,27–49^. The network geometry is shown in Supplemental Information Fig. 3. Patients on ascorbic acid alone, or the combination of glucocorticoid plus ascorbic acid, or the combination of glucocorticoid and fludrocortisone had lower short-term mortality (Fig. 1). Forest plot for components showed the use of ascorbic acid was associated with lower short-term mortality (Supplementary Fig. 4). There was no evidence of statistical heterogeneity (τ^2^ = 0, p = 927). The designment-by-treatment interaction model and node-splitting method did not detect any inconsistencies (Supplemental Information Table 17). There was no evidence of publication bias based on the funnel plot and Egger’s test (Supplemental Information Fig. 5).

### Secondary outcomes

#### Longer term mortality

Seven studies including 6,322 patients presented the longer-term mortality (≥ 90 days)^1,3,28,36,37,45,49^. The network geometry is shown in Supplemental Information Fig. 6. The combination of glucocorticoid and fludrocortisone was associated with increased longer-term mortality compared with placebo (RR 0.89, 95% CI 0.82–0.98) (Supplemental Information Fig. 7). There was no statistically significant difference of individual components compared with placebo in reducing longer-term mortality (Supplementary Fig. 8).

#### Time to resolution of shock

Fourteen studies including 5063 patients presented the time to resolution of shock^1,2,23,32–35,37,39,41–44,47^. The network geometry is shown in Supplemental Information Fig. 9. Both glucocorticoid and the combination of glucocorticoid, ascorbic acid and thiamine shortened the time to resolution of shock compared with placebo (Fig. 2). Forest plot for components showed glucocorticoid shortened the time to resolution of shock with statistical significance (Supplemental Information Fig. 10) There was no evidence of publication bias (Supplemental Information Fig. 11).

#### Duration of mechanical ventilation

Based on the six studies which presented the duration of mechanical ventilation (network geometry as shown in Supplemental Information Fig. 12)^2,33,34,37,45,47^, patients on glucocorticoid had shorter duration of mechanical ventilation compared with placebo (Supplemental Information Fig. 13). Forest plot for components showed glucocorticoid shortened the duration of mechanical ventilation compared with placebo (Supplemental Information Fig. 14).

### Tertiary outcomes

#### ICU and hospital length of stay

Network geometries on ICU and hospital stay were shown in Supplemental Information Fig. 15 and 16 respectively. There was no significant difference in ICU length of stay based on the data from 10 studies^3,30–33,35,37,39,44,45^ (Supplemental Information Fig. 17). Based on the 9 studies presenting hospital length of stay^3,6,30,31,33,35,42,44,45^, there was also no statistically significant difference in hospital length of stay (Supplemental Information Fig. 18) There was no evidence of publication bias in ICU length of stay (Supplementary Fig. 19).

#### Adverse events

The use of glucocorticoid or the combination of glucocorticoid and fludrocortisone was associated with increased risk of hyperglycemia. There was no difference in the incidence of secondary infections, gastrointestinal bleeding, delirium, and hypernatremia with the use of steroid as shown in the pooled data (Supplemental Information Fig. 20). One study reported the incidence of hypernatremia that was not amenable to statistical pooling, showing the lower risk of hypernatremia with hydrocortisone infusion compared with bolus injection^50^. Adverse events related to ascorbic acid or thiamine were uncommon (Supplemental Information Table 18).

### Sensitivity analysis

Sensitivity analysis performed on short-term mortality limiting to studies adjudicated with low risk of bias^3,4,6,30,33,34,36–38,40,45,49^ (Supplemental Information Fig. 21) and studies recruiting > 75% patients dependent on inotrope/ vasopressor^1,3,4,6,19,23,27,28,30–38,40–47^ showed similar results (Supplemental Information Fig. 22). In the sensitivity analysis on studies recruiting patients after 2016^6,37–49^, the use of ascorbic acid did not reach statistical significance in additive CNMA and there was no data on the use of glucocorticoid and fludrocortisone (Supplemental Information Fig. 23). Sensitivity analysis performed on studies excluding high-dose glucocorticoid (≥ 400 mg/day hydrocortisone or equivalent) showed similar results (Supplemental Information Fig. 24–26)^1,3,4,6,23,27–49^. None of studies reporting longer term mortality used high dose corticosteroid so sensitivity analysis was omitted.

### P-score statistics on primary and secondary outcomes

Table 1 shows the P-scores of each intervention on short-term mortality, longer-term mortality, and time to resolution of shock. Ascorbic acid was ranked as the best treatment for short term mortality, whereas glucocorticoid plus fludrocortisone was ranked the first for longer-term mortality. Glucocorticoid was ranked the best in hastening the resolution of shock.

**Table 1:** P-score statistics.

	GLU	AA	THI	GLU + AA	GLU + FLU	GLU + AA + THI	AA + THI	Placebo
Short-term mortality	0.451	0.926	0.292	0.727	0.693	0.366	0.195	0.351
Longer-term mortality	0.539	–	–	–	0.962	0.264	0.236	0.500
Time to resolution of shock	0.716	0.359	0.140	0.864	–	0.713	–	0.208

*GLU* Glucocorticoid, *AA* ascorbic acid, *THI* Thiamine, *FLU* Fludrocortisone.

## Discussion

The results of this NMA of 34 RCT offered evidence summary regarding the use of steroid, ascorbic acid, and thiamine in adult patients with sepsis and septic shock. Over the years, the interest of the researchers on sepsis changed from high-dose corticosteroid alone, to low dose corticosteroid, and to combination with fludrocortisone, and then to various septic cocktails. There were limited trials with head-to-head comparisons. Worries about their potential complications might limit the choice among these septic adjuncts as well. Clinicians would be eager to know the effect of individual component from the septic cocktail and the clinical benefits of each combination therapy. In this NMA, we found that two interventions were associated with improved short-term mortality, namely ascorbic acid and the combination of glucocorticoid and fludrocortisone. Only the effect of the latter translated into improved longer- term mortality. Glucocorticoid shortened the time to resolution of shock and the duration of mechanical ventilation, but not the ICU or hospital length of stay. Complications arising from these interventions were uncommon except for hyperglycemia associated with steroid use.

This systematic review had a number of strengths. While there had been systematic reviews evaluating the use of steroids, or thiamine in patients with septic shock^51,52^, this systematic review looked at the use of steroids, ascorbic acid, and thiamine together as an NMA. This study went one step further to dismantle the effect from individual components among various combinations of steroids, and ascorbic acid by CNMA. Another strength of this study was the independent risk of bias assessment where the reviewers were not involved in any of the included trials. There was comprehensive literature search and adherence to the PRISMA-NMA guideline.

Obviously, this meta-analysis could not address all the unresolved issues in the use of these adjunct therapy in the management of septic shock and there are several limitations in this study. Drawing a conclusion in meta-analysis is dependent on the availability and quality of the exist trials. The very assumption underlying a meta-meta-analysis is transitivity. While we believed that the patient characteristics and the use of steroid were reasonably comparable among studies, the understanding of diseases, ICU management strategy and the definition of sepsis and septic shock have been changing over the last thirty plus years when the recruited studies have been conducted. Ideally there should be head-to-head comparison between hydrocortisone, ascorbic acid, thiamine, septic cocktail, and the combination of hydrocortisone plus fludrocortisone. However, in practice, with the existing evidence and established guideline supporting the use of hydrocortisone, conducting another randomized controlled trial with ascorbic acid alone or thiamine alone might be contrary to the principle of equipoise.

Despite the solid evidence on the use of glucocorticoid and fludrocortisone and a flourishing number of papers focusing the use of ‘septic cocktail’, guidelines on sepsis management such as the Surviving Sepsis Campaign have been slow to recognize the role of these agents. Since the publication of the two ‘positive’ trials form Annane^28,36^, no guidelines have been published to adopt such regimen. Based on the results from our NMA, the role of glucocorticoid and fludrocortisone should be re-emphasized, although further research is needed to clarify the role of glucocorticoid plus fludrocortisone in the era post-2016 Surviving Sepsis Campaign and its effect as compared with the septic cocktail. On the other hand, the results of the current NMA did not support the routine use of thiamine and ascorbic acid.

## Conclusions

In adults with sepsis and septic shock, the combination of glucocorticoid and fludrocortisone improved short-term and longer-term mortality. Glucocorticoid shortened the time to resolution of shock and duration of mechanical ventilation but increased incidence of hyperglycemia. There was no strong evidence supporting the routine use of thiamine and ascorbic acid, but they were associated with minimal adverse effects.

## Supplementary Information

Supplementary Information 1.

Supplementary Information 2.

## Data Availability

All data generated or analyzed during the present study are included in this published article and its supplementary information files.
