# Supplementary material for: Steroid, ascorbic acid, and thiamine in adults with sepsis and septic shock: a systematic review and component network meta-analysis
Source: Sci Rep. 2021 Aug 4;11:15777. doi: 10.1038/s41598-021-95386-9 (PMC8338943; doi:10.1038/s41598-021-95386-9)
Supplement: Supplementary file 1 — Supplementary Information 1. [file 41598_2021_95386_MOESM1_ESM.docx]

**Supplementary Information**

**Steroid, ascorbic acid, and thiamine in adults with sepsis and septic shock: a systematic review and component network meta-analysis**

Ka Man Fong, Shek Yin Au, George Wing Yiu Ng

Table of content

[Table 1. PRISMA-NMA Checklist 4](#_Toc73002258)

[Table 2. PubMed Search Strategy 8](#_Toc73002259)

[Table 3. Characteristics of the included studies 9](#_Toc73002260)

[Table 4. Results of individual studies on short-term mortality (<90 days) 24](#_Toc73002261)

[Table 5. Results of individual studies on longer-term mortality (≥90 days) 27](#_Toc73002262)

[Table 6. Results of individual studies on time to resolution of shock 28](#_Toc73002263)

[Table 7. Results of individual studies on duration of mechanical ventilation 29](#_Toc73002264)

[Table 8. Results of individual studies on ICU length of stay 30](#_Toc73002265)

[Table 9. Results of individual studies on hospital length of stay 31](#_Toc73002266)

[Table 10. Results of individual studies on secondary infections 32](#_Toc73002267)

[Table 11. Results of individual studies on gastrointestinal bleeding 33](#_Toc73002268)

[Table 12. Results of individual studies on delirium 34](#_Toc73002269)

[Table 13. Results of individual studies on hyperglycemia 35](#_Toc73002270)

[Table 14. Results of individual studies on hypernatremia 36](#_Toc73002271)

[Table 15. Investigation of transitivity assumption 37](#_Toc73002272)

[Table 16. Direct, indirect, and network meta-analysis estimates of glucocorticoid, ascorbic acid, and thiamine in patients with sepsis and septic shock on short term mortality 39](#_Toc73002273)

[Table 17. Inconsistency tests for short-term mortality 41](#_Toc73002274)

[Table 18. Adverse events reported by studies potentially related to ascorbic acid or thiamine 42](#_Toc73002275)

[Figure 1. PRISMA flow diagram of the search results 43](#_Toc73002276)

[Figure 2. Summary of risk of bias assessment 44](#_Toc73002277)

[Figure 3. Network geometry of studies presenting short-term mortality (<90 days) 45](#_Toc73002278)

[Figure 4. Forest plot for components on short-term mortality (<90 days) 46](#_Toc73002279)

[Figure 5. Funnel plot of network meta-analysis on short-term mortality (<90 days) 47](#_Toc73002280)

[Figure 6. Network geometry of studies presenting longer-term mortality (≥ 90 days) 48](#_Toc73002281)

[Figure 7. Network meta-analysis on longer-term mortality (≥ 90 days) 49](#_Toc73002282)

[Figure 8. Forest plot for components on longer-term mortality (≥ 90 days) 50](#_Toc73002283)

[Figure 9. Network geometry of studies presenting time to resolution of shock 51](#_Toc73002284)

[Figure 10. Forest plot for components in time to resolution of shock 52](#_Toc73002285)

[Figure 11. Funnel plot on time to resolution of shock 53](#_Toc73002286)

[Figure 12. Network geometry on duration of mechanical ventilation 54](#_Toc73002287)

[Figure 13. Network meta-analysis on duration of mechanical ventilation 55](#_Toc73002288)

[Figure 14. Forest plot for components on duration of mechanical ventilation 56](#_Toc73002289)

[Figure 15. Network geometry on ICU length of stay 57](#_Toc73002290)

[Figure 16. Network geometry on hospital length of stay 58](#_Toc73002291)

[Figure 17. Network meta-analysis on ICU length of stay 59](#_Toc73002292)

[Figure 18. Network meta-analysis on hospital length of stay 60](#_Toc73002293)

[Figure 19. Funnel plot on ICU length of stay 61](#_Toc73002294)

[Figure 20. Adverse events related to steroid 62](#_Toc73002295)

[Figure 21. Sensitivity analysis of network meta-analysis on short-term mortality limiting to studies of low risk of bias 63](#_Toc73002296)

[Figure 22. Sensitivity analysis of network meta-analysis on short-term mortality limiting to studies recruiting >50% of patients dependent on inotrope/ vasopressor 64](#_Toc73002297)

[Figure 23. Sensitivity analysis of network meta-analysis on short-term mortality limiting to studies recruiting patients after 2016 65](#_Toc73002298)

[Figure 24 Sensitivity analysis of network meta-analysis on short-term mortality excluding studies using high dose corticosteroid (≥400mg/day hydrocortisone or equivalent) 66](#_Toc73002299)

[Figure 25 Sensitivity analysis of network meta-analysis on time to resolution of shock excluding studies using high dose corticosteroid (≥400mg/day hydrocortisone or equivalent) 67](#_Toc73002300)

[Figure 26 Sensitivity analysis of network meta-analysis on time of mechanical ventilation excluding studies using high dose corticosteroid (≥400mg/day hydrocortisone or equivalent) 68](#_Toc73002301)

Table 1. PRISMA-NMA Checklist

**PRISMA NMA Checklist of Items to Include When Reporting A Systematic Review Involving a Network Meta-analysis**

| **Section/Topic** | **Item #** | **Checklist Item** | **Reported on Page #** |
| --- | --- | --- | --- |
| **TITLE** |  |  |  |
| Title | 1 | Identify the report as a systematic review *incorporating a network meta-analysis (or related form of meta-analysis).* | ***1*** |
|  |  |  |  |
| **ABSTRACT** |  |  | ***2*** |
| Structured summary | 2 | Provide a structured summary including, as applicable:  **Background:** main objectives  **Methods:** data sources; study eligibility criteria, participants, and interventions; study appraisal; and *synthesis methods, such as network meta-analysis.*  **Results:** number of studies and participants identified; summary estimates with corresponding confidence/credible intervals; *treatment rankings may also be discussed. Authors may choose to summarize pairwise comparisons against a chosen treatment included in their analyses for brevity.*  **Discussion/Conclusions:** limitations; conclusions and implications of findings.  **Other:** primary source of funding; systematic review registration number with registry name. |  |
|  |  |  |  |
| **INTRODUCTION** |  |  |  |
| Rationale | 3 | Describe the rationale for the review in the context of what is already known*, including mention of why a network meta-analysis has been conducted.* | ***4*** |
| Objectives | 4 | Provide an explicit statement of questions being addressed, with reference to participants, interventions, comparisons, outcomes, and study design (PICOS). | 4 |
|  |  |  |  |
| **METHODS** |  |  |  |
| Protocol and registration | 5 | Indicate whether a review protocol exists and if and where it can be accessed (e.g., Web address); and, if available, provide registration information, including registration number. | 5 |
| Eligibility criteria | 6 | Specify study characteristics (e.g., PICOS, length of follow-up) and report characteristics (e.g., years considered, language, publication status) used as criteria for eligibility, giving rationale. *Clearly describe eligible treatments included in the treatment network, and note whether any have been clustered or merged into the same node (with justification).* | ***5*** |
| Information sources | 7 | Describe all information sources (e.g., databases with dates of coverage, contact with study authors to identify additional studies) in the search and date last searched. | 5 |
| Search | 8 | Present full electronic search strategy for at least one database, including any limits used, such that it could be repeated. | Supplementary Information Table 2 |
| Study selection | 9 | State the process for selecting studies (i.e., screening, eligibility, included in systematic review, and, if applicable, included in the meta-analysis). | 5 |
| Data collection process | 10 | Describe method of data extraction from reports (e.g., piloted forms, independently, in duplicate) and any processes for obtaining and confirming data from investigators. | 5 |
| Data items | 11 | List and define all variables for which data were sought (e.g., PICOS, funding sources) and any assumptions and simplifications made. | 5 |
| **Geometry of the network** | **S1** | Describe methods used to explore the geometry of the treatment network under study and potential biases related to it. This should include how the evidence base has been graphically summarized for presentation, and what characteristics were compiled and used to describe the evidence base to readers. | *7* |
| Risk of bias within individual studies | 12 | Describe methods used for assessing risk of bias of individual studies (including specification of whether this was done at the study or outcome level), and how this information is to be used in any data synthesis. | 6 |
| Summary measures | 13 | State the principal summary measures (e.g., risk ratio, difference in means). *Also describe the use of additional summary measures assessed, such as treatment rankings and surface under the cumulative ranking curve (SUCRA) values, as well as modified approaches used to present summary findings from meta-analyses.* | 7 |
| Planned methods of analysis | 14 | Describe the methods of handling data and combining results of studies for each network meta-analysis. This should include, but not be limited to:   - *Handling of multi-arm trials;* - *Selection of variance structure;* - *Selection of prior distributions in Bayesian analyses; and* - *Assessment of model fit.* | 7 |
| **Assessment of Inconsistency** | **S2** | Describe the statistical methods used to evaluate the agreement of direct and indirect evidence in the treatment network(s) studied. Describe efforts taken to address its presence when found. | 7 |
| Risk of bias across studies | 15 | Specify any assessment of risk of bias that may affect the cumulative evidence (e.g., publication bias, selective reporting within studies). | **7** |
| Additional analyses | 16 | Describe methods of additional analyses if done, indicating which were pre-specified. This may include, but not be limited to, the following:   - Sensitivity or subgroup analyses; - Meta-regression analyses; - *Alternative formulations of the treatment network; and* - *Use of alternative prior distributions for Bayesian analyses (if applicable).* | ***7*** |
|  |  |  |  |
| **RESULTS†** |  |  |  |
| Study selection | 17 | Give numbers of studies screened, assessed for eligibility, and included in the review, with reasons for exclusions at each stage, ideally with a flow diagram. | 8, Supplemental Information Figure 1 |
| **Presentation of network structure** | **S3** | Provide a network graph of the included studies to enable visualization of the geometry of the treatment network. | *Supplemental Information Figure 3,6,9,12,15,16* |
| **Summary of network geometry** | **S4** | Provide a brief overview of characteristics of the treatment network. This may include commentary on the abundance of trials and randomized patients for the different interventions and pairwise comparisons in the network, gaps of evidence in the treatment network, and potential biases reflected by the network structure. | *Supplemental Information Figure 3,6,9,12,15,16* |
| Study characteristics | 18 | For each study, present characteristics for which data were extracted (e.g., study size, PICOS, follow-up period) and provide the citations. | *Supplemental Information Table 3* |
| Risk of bias within studies | 19 | Present data on risk of bias of each study and, if available, any outcome level assessment. | *Supplemental Information Figure 2* |
| Results of individual studies | 20 | For all outcomes considered (benefits or harms), present, for each study: 1) simple summary data for each intervention group, and 2) effect estimates and confidence intervals. *Modified approaches may be needed to deal with information from larger networks.* | Supplemental Information Table 4 - 14 |
| Synthesis of results | 21 | Present results of each meta-analysis done, including confidence/credible intervals. *In larger networks, authors may focus on comparisons versus a particular comparator (e.g. placebo or standard care), with full findings presented in an appendix. League tables and forest plots may be considered to summarize pairwise comparisons.* If additional summary measures were explored (such as treatment rankings), these should also be presented. | 9-13  Figure 1,2, Supplemental Information Figure 7, 9, 10, 13, 14, 17, 18 |
| **Exploration for inconsistency** | **S5** | Describe results from investigations of inconsistency. This may include such information as measures of model fit to compare consistency and inconsistency models, *P* values from statistical tests, or summary of inconsistency estimates from different parts of the treatment network. | *Supplemental Information Table 16* |
| Risk of bias across studies | 22 | Present results of any assessment of risk of bias across studies for the evidence base being studied. | Supplemental Information Figure 2 |
| Results of additional analyses | 23 | Give results of additional analyses, if done (e.g., sensitivity or subgroup analyses, meta-regression analyses*, alternative network geometries studied, alternative choice of prior distributions for Bayesian analyses,* and so forth). | *13-14*  *Supplemental Information Figure 21-26* |
|  |  |  |  |
| **DISCUSSION** |  |  |  |
| Summary of evidence | 24 | Summarize the main findings, including the strength of evidence for each main outcome; consider their relevance to key groups (e.g., healthcare providers, users, and policy-makers). | 14-15 |
| Limitations | 25 | Discuss limitations at study and outcome level (e.g., risk of bias), and at review level (e.g., incomplete retrieval of identified research, reporting bias). *Comment on the validity of the assumptions, such as transitivity and consistency. Comment on any concerns regarding network geometry (e.g., avoidance of certain comparisons).* | 15-16 |
| Conclusions | 26 | Provide a general interpretation of the results in the context of other evidence, and implications for future research. | 17 |
|  |  |  |  |
| **FUNDING** |  |  | 18 |
| Funding | 27 | Describe sources of funding for the systematic review and other support (e.g., supply of data); role of funders for the systematic review. This should also include information regarding whether funding has been received from manufacturers of treatments in the network and/or whether some of the authors are content experts with professional conflicts of interest that could affect use of treatments in the network. |  |

PICOS = population, intervention, comparators, outcomes, study design.

* Text in italics indicates wording specific to reporting of network meta-analyses that has been added to guidance from the PRISMA statement.

† Authors may wish to plan for use of appendices to present all relevant information in full detail for items in this section.

Table 2. PubMed Search Strategy

|  | Searches |
| --- | --- |
| #1 | Vasopressor OR vasopressor dependence OR septicemia OR septic shock OR sepsis |
| #2 | (((((clinical trial as topic[MeSH Terms]) OR (randomized controlled trial[Publication Type])) OR (controlled clinical trial[Publication Type])) OR (trial[Title/Abstract])) OR (random[Title/Abstract])) |
| #3 | (((hydrocortisone OR glucocorticoid OR adrenal cortex hormones OR corticosteroids OR fludrocortisone OR dexamethasone OR methylpred* OR betamethasone)) OR (ascorbic acid OR ascorbate OR vitamin C) OR (thiamine OR vitamin B1)) |
| #4 | #1 AND #2 AND #3 |

Table 3. Characteristics of the included studies

| Study and published year | Countries; No. of centers; Settings | Definition of sepsis/ septic shock | APACHE  II | Onset of septic shock | First Intervention | Second intervention | Third Intervention | Key outcomes |
| --- | --- | --- | --- | --- | --- | --- | --- | --- |
| Sprung 1984 [19] | United States; 2; Medical ICU | Septic shock (1) SBP < 90mmHg or 50mmHg less than a previously defined pressure in a hypertensive patient, (2) decreased organ perfusion as evidenced by altered mental status or oliguria (<20ml/hour), (3) continued hypotension despite an intravenous infusion of at least 500ml of NS, and (4) bacteremia or an identified source of infection | NR | NR | MP 30mg/kg infusion up to 2 doses (n=21) | DX 6mg/kg up to 2 doses (n=22) | No corticosteroid  (n=16) | Reversal of shock and survival to hospital discharge |
| Bone 1987 [20] | United Sates; 19; NR | Clinical diagnosis of sepsis (clinical evidence of infection, fever >38.3°C or hypothermia <35.6°C, tachycardia > 90 bpm, tachypnea > 20bpm, at least one of the following signs of inadequate organ perfusion/ organ, or septic shock: sustained decrease in SBP to <90mmHg, or a drop of 40mmHg from baseline, for at least one hour with adequate volume replacement and patient not taking anti-hypertensives | NR | < 2 hours | MP 30mgkg Q6H for 4 doses (n=191) | Placebo (n=191) | - | development of septic shock within 14 days of study admission in patients not in shock at entry; reversal of septic shock within 14 days of study admission, death within 14 days of study admission |
| Luce 1987 [21] | United States; 2; Medical and surgical ICUs | (1) already hospitalized patient with a known onset of presumed septic shock, who manifested within 4 hours of last observation in ICU an increase in body temperature of ≥ 1.5°C, and decrease in SBP of ≥20mmHg from baseline , or (2) patients with an unknown time of onset of presumed septic shock, most of whom were admitted directly from the ED or OR, who manifested a temperature >38.5 °C or < 35.5°C or SBP <90mmHg | NR | Hospitalized patients < 4 hours, or unknown onset from ED/ OT | MP 30mg/kg Q6H for 4 doses (n=38) | Placebo (n=37) | - | Development and reversal of ARDS |
| Hinshaw 1987 [22] | United States; 10; NR | Clinical suspicion of sepsis and who had at least 4 of the following 7 clinical signs of sepsis within 8 hour period (1) shaking chills or fever (temperature > 38.9°C) or both, or hypothermia (<35.5°C), (2) tachypnea (>28bpm) or hypocapnia (PaCO2<32mmHg), (3) tachycardia (HR>100bpm), (4) hypotension (SBP<90mmHg), (5) abnormal WCC <3500 or ≥15000 per cubic millimeter, abnormal neutrophil count (<35 or >85% of total white cell count), or abnormal immature neutrophil (Band) count (>20% of total white cell count), (6) thrombocytopenia (<100,000 platelets per cubic millimeter), (7) surgical or invasive procedure performed during the preceding 48 hours or the presence of an obvious primary septic site | NR | <8 hours | MP 30kg/kg bolus, then 5mg/kg for 9 hours (n=112) | Placebo (n=111) | - | 14-day mortality from all causes |
| Bollaert 1998 [27] | France; 2; ICU | ACCP/SCCM criteria and received vasopressor therapy for >48hours; | NR | > 48 hours | HC 100mg Q8H for 5 days, if shock reversal, continued with half doses for 3 days, then quarter doses for 3 days (n=22) | Placebo (n=19) | - | Shock reversal  Blood lactate <2mmol/L |
| Briegel 1999 [1] | Germany; 1; ICU | ACCP/SCCM criteria for septic shock, on vasopressor support and high output circulatory failure (CI>4L/min/m^2^) after fluid resuscitation and without the use of positive inotropes such as dobutamine or dopexamine | HC 26 ± 1,  Placebo 27 ± 1 | < 72 hours | HC 100mg in 30min, then 0.18mg/kg/hour and taper off on cessation of vasopressor  (n=20) | Placebo (n=20) | - | Time to cessation of vasopressor support |
| Annane 2002 [28] | France; 19; ICU | 1) documented site (or at least strong suspicion) of infection, as evidenced by one or more of the following: presence of polymorphs in a normally sterile body fluid, positive culture or Gram stain of a normally sterile body fluid, clinical focus of infection, wound with purulent discharge, pneumonia or other clinical evidence of systemic infection; AND 2) temperature > 38.3°C or <35.6°C, AND 3) heart rate greater than 90bpm, AND 4) SBP<90mmgHg for at least 1 hour despite adequate fluid replacement and more than 5 microgram/kg of body weight of dopamine or current treatment with epinephrine or norepinephrine, AND 5) urinary output < 0.5ml/kg of body weight for at least 1 hour or PaO2/FiO2 < 280mmHg, AND 6) arterial lactate > 2mmol/L, AND 7) need for mechanical ventilation | NR | < 3 hours | HC 50mg Q6H, FLU 50 microgram daily for 7 days (n=150) | Placebo  (n=149) | - | 28-day survival distribution from randomization in non-responders to short corticotrophin test |
| Oppert 2005 [23] | Germany; 1; Medical ICU | 1) two or more of the following: tachycardia > 90bpm, temperature ≥ 38.5°C or < 36°C, leucocytosis ≥12000/nL or ≥10% immature cells, respiratory rate >20bpm or mechanical ventilation; 2) evidence or strong clinical suspicion of infection, positive culture or gram negative stain of a normally sterile body fluid, clinical focus of infection, 3) SBP<90mmHg for ≥ 1 hour despite adequate fluid resuscitation as demonstrated by a CVP≥ 10mmHg and/or PAOP≥ 15mmHg, 4) CI≥ 3.5L/min/m^2^, and 5) need for vasopressor support | HC 25 [19-30]  Placebo 25.5 [19.8-29] | < 24 hours | HC 50mg bolus, then 0.18mg/kg/hour, taper off on cessation of vasopressor (n=18) | Placebo (n=23) | - | Time to cessation of vasopressor support |
| Cicarelli 2007 [2] | Brazil; 1; Surgical ICU | NR | DX 20 ± 5,  Placebo 19 ± 4 | NR | DX 0.2mg/kg, 3 doses interval of 36 hours (n=15) | Placebo (n=14) | - | Duration of vasopressor support, duration of mechanical ventilation, mortality |
| Sprung 2008 [3] | Austria, Belgium, France, Germany, Israel, Italy, Netherlands, Portugal, United Kingdom; 52; ICU | Clinical evidence of infection, and onset of shock within the previous 72 hours (SBP<90mmHg despite fluid replacement or a need for vasopressor for ≥ 1 hour) and hypoperfusion or organ dysfunction attributable to sepsis | HC 63 ±14  Placebo 63 ± 15 | < 72 hours | HC 50mg Q6H for 5 days, then 50mg Q12H for 3 days, then 50mg Q24H for 3 days (n=251) | Placebo (n=248) | - | Rate of death at 28 days in patient who did not have a response to corticotropin |
| Meduri 2009 [29] | NR; NR; ICU | NR | NR | NR | HC 10mg/hour for 7 days(n=48) | Placebo (n=32) | - | Median day 7 CRP, duration of mechanical ventilation, ICU and hospital survival, ICU duration |
| Annane 2010 [30] | France; 11; ICU | (1) criteria for severe sepsis as defined by ACCP/SCCM, (2) MOD as defined by SOFA≥ 8, (3) need for vasopressor therapy to maintain SBP≥ 90mmHg or MAP≥ 60mmHg, and 4) receiving 50mg HC IV Q6H as an adjunct therapy for septic shock | NR | NR | HC 50mg Q6H, FLU 50 microgram daily for 7 days (n=245) | HC 50mg Q6H (n=264) | - | In-hospital mortality (or 90-day mortality whichever occurred first) |
| Gordon 2014 [4] | United Kingdom; 4; ICU | 2 out of 4 SIRS due to known or suspected infection, and who required vasopressors despite adequate IV fluid resuscitation | HC 19 [14-22] ,  Placebo 20 [17-25] | NR | Vasopressin and HC 50mg Q6H for 5 days, then 50mg Q12H for 3 days, then 50mg Q24H for 3 days (n=31) | Vasopressin and Placebo (n=30) | - | Change in plasma vasopressin concentrations |
| Mirea 2014 [49] | Romania; NR; NR | NR | NR | NR | HC 200mg/day in 4 doses (n=58) | HC 200mg/day infusion (n=59) | No HC (n=54) | Sodium level |
| Donnino 2016 [31] | United States; 2; ED and ICU | Presence of two of more SIRS criteria with documented or suspected infection), lactate>3mmol/L, hypotension (SBP<90mmHg) after ≥ 2L fluid bolus followed by vasopressor-dependence | THI 25.7 ± 9.1,  Placebo 26.5 ± 9.2 | NR | THI 200mg Q12H for 7 days (n=43) | Placebo (n=45) | - | Lactate level 24 hours after first study medication dose |
| Zabet 2016 [32] | Iran; 1; ICU | SSC criteria of septic shock, who needed a vasopressor drug to maintain MAP>65mmHg despite fluid resuscitation | AA 19.1 ± 5.2, Placebo 23.0 ± 5.6 | NR | AA 25mg/kg Q6H for 72 hours (n=14) | Placebo (n=14) | - | Vasopressor dose and duration |
| Gordon 2016 [33] | United Kingdom; 18; ICU | Sepsis (2 of 4 SIRS criteria due to known or suspected infection) and who required vasopressors despite adequate IVF resuscitation | 24 [19-30] | < 6 hours | HC 50mg Q6H for 5 days, then 50mg Q12H for 3 days, then 50mg Q24H for 3 days (n=158) | Placebo (n=164) |  | Kidney failure free days during the 28 days |
| Tongyoo 2016 [34] | Thailand; 1; Medical ICU | ACCP/SCCM criteria for severe sepsis or septic shock | HC 21.5 ± 5.7, Placebo 21.9 ± 5.7 | NR | HC 50mg Q6H for 7 days (n=98) | Placebo (n=99) | - | All-cause mortality at study day 28 |
| Lv 2017 [35] | China; 1; ICU | NR | HC 25.5 ± 9.5, Placebo 21.3 ± 6.9 | < 6 hours | HC 200mg infusion for 6 days and taper off  (n=58) | Placebo (n=60) | - | 28-day mortality |
| Annane 2018 [36] | France; 34; ICU | Indisputable/ probable septic shock for <24 hours (clinically or microbiologically documented infection, a SOFA 3 or 4 for ≥2 organs and ≥6 hours, receipt of vasopressor therapy for ≥6 hours to maintain SBP> 90mmHg or MAP>65mmHg | NR | > 6 hours | HC 50mg Q6H, FLU 50 microgram daily for 7 days (n=614) | Placebo (n=627) | - | 90-day all-cause mortality |
| Venkatesh 2018 [37] | Australia, United Kingdom, New Zealand, Saudi Arabia, Denmark; 69; ICU | ≥2 criteria of the SIRS, and who had been treated with vasopressors or inotropic agents for ≥ 4 hours | HC 24.0 [19.0-29.0], Placebo 23.0 [18.0-29.0] | > 4 hours | HC 200mg/day infusion for 7 days (n=1853) | Placebo (n=1860) | - | Death from any cause at 90 days after randomisation |
| Fowler 2019 [38] | United States; 7; Medical ICU | Suspected or proven infection, and met 2 of 4 SIRS | NR | NR | AA 50mg/kg Q6H for 96 hours (n=84) | Placebo (n=83) | - | mSOFA at 96 hours and plasma biomarkers (CRP, thrombomodulin) at 168 hours |
| Harun 2019 [39] | Kuala Lumpur; 1; ICU | Septic shock (Presence of ≥2 SIRS criteria with documented or suspected infection, hypotension requiring use of vasopressors to maintain MAP ≥2, and having serum lactate ≥2mmol/L despite adequate fluid resuscitation) | THI 35.0 [28.3-37.8], Placebo 29.0 [23.0-34.0] | NR | THI 200mg Q8H for 3 days (n=32) | Placebo (n=33) | - | Relative lactate changes over 24 hours, time for shock reversal, relative changes of SOFA over 72 hours, ICU LOS, ICU mortality |
| Fujii 2020 [6] | Australia, New Zealand, Brazil; 10; ICU | Sepsis-3 criteria: suspected or documented infection with an acute increase of ≥2 points SOFA, had a lactate > 2mmol/L, and were vasopressor dependent for ≥ 2 hours | NR | <24 hour | HC 50mg Q6H, AA 1.5g Q6H, THI 200mg Q12H  (n=107) | HC 50mg Q6H (n=104) | - | Time alive and free of vasopressors at day 7 after randomization |
| Moskowitz 2020 [40] | United States; 14; ICU | Suspected or confirmed infection and were receiving a vasopressor because of sepsis | NR | <24 hour | HC 50mg Q6H, AA 1.5g Q6H, THI 100mg Q6H for 4 days  (n=101) | Placebo (n=99) | - | Change in SOFA between enrolment and 72-hour follow-up |
| Iglesias 2020 [41] | United States; 2; ICU | Primary diagnosis of sepsis or septic shock according to the 2016 SSC, diagnosis of sepsis or septic shock within 12 hours | HC+AA+THI 24±7.6, Placebo 24.9 ± 8.7 | <12 hour | HC 50mg Q6H, AA 1.5g Q6H, THI 200mg Q12H for maximum 4 days (n=68) | Placebo (n=69) | - | Resolution of shock and change in SOFA score |
| Wani 2020 [42] | India; 1; Department of Internal Medicine and ED | Sepsis-3 criteria | HC+AA+THI 18.5 [15-24.75], Placebo 20[15-24] | NR | HC 50mg Q6H for 7 days, AA 1.5g Q6H for 4 days, THI 200mg Q12H for 4 days (n=50) | Placebo (n=50) |  | In-hospital mortality |
| Reddy 2020 [43] | India; 1; ICU | Rise in SOFA of 2 with persistent hemodynamic instability despite fluid resuscitation and requiring vasopressor | HC+AA+THI 18.4±2.5, HC+AA 17 ± 3.8, HC 21.2±6.5 | NR | HC 200mg over 24-hour infusion, AA 1.5g Q6H, THI 200mg Q12H (n=9) | HC 200mg over 24-hour infusion, AA 1.5g Q6H (n=9) | HC 200mg over 24-hour infusion (n=9) | Time to shock reversal |
| Mohamed 2020 [44] | India; 1; ICU | SSC criteria | NR | < 6 hour | HC 50mg Q6H, AA 1.5g Q6H, THI 200mg Q12H (n=45) | Placebo (n=43) | - | Inpatient all-cause mortality |
| Hwang 2020 [45] | South Korea; 4; ED | Diagnosis of sepsis with persistent hypotension requiring vasopressors to maintain MAP≥65mmHg and having serum lactate >2mmol/L despite adequate fluid challenge | AA + THI 22 [14-32], Placebo 22 [17-32] | < 24 hour | AA 50mg/kg Q12H , THI 200mg Q12H for 48 hours (n=53) | Placebo (n=58) | - | SOFA score change at 72 hour |
| Petsakul 2020 [46] | Thailand; 1; ICU | Suspected infection, SOFA≥2, on a vasopressor / inotropic drug and a lactate level >2mmol/L | THI 26 ± 7.6, Placebo 29 ± 6.1 | > 1 hour and < 24 hour | THI 200mg Q12H for 7 days  (n=25) | Placebo (n=25) | - | Vasopressor-free days over 7 days |
| Chang 2020 [47] | China; 1; ICU | Sepsis-3 and Procalcitonin ≥2ng/ml | HC+AA+THI 22.1 ± 8.4, Placebo 23.8 ± 7.6 | NR | HC 50mg Q6H for 7 days, AA 1.5g Q6H for 4 days, THI 200mg Q12H for 4 days  (n=40) | Placebo (n=40) | - | All-cause mortality within 28 days |
| Aisa-Alvarez 2020 [48] | Mexico; 2; ICU | Sepsis-3, acute increase of ≥ 2 points in SOFA, lactate >2mmol/L, vasopressor dependent | NR | ≥ 2 hour and < 24 hour | AA 1mg Q6H Nasogastric (n=18) | No antioxidant (n=21) | - | Change in SOFA scores in 5 days |
| Sevransky 2021 [49] | United States; 43; ICU | Cardiovascular dysfunction caused by suspected infection with planned ICU admission;  Infection: ordering of blood cultures and administration of ≥1 antimicrobial agent);  Cardiovascular dysfunction: need for any vasopressor for ≥1 hour to maintain MAP≥65 despite ≥1L IV crystalloids | HC+AA+THI 27 [22-33], Placebo 27 [19-33] | < 24 hour | HC 50mg, AA 1.5g, THI 100mg Q6H up to 96 hours (n=252) | Placebo (n=249) | - | Ventilator and vasopressor free days in the first 30 days |

ICU, Intensive Care Unit; bpm: breath per minute/ beat per minute; NR, Not reported; ED, Emergency Department; OT, Operating Theatre; OR, Operating room; SBP, Systolic blood pressure; MAP, mean arterial pressure; SOFA, modified Sequential Organ Failure Assessment score; mSOFA, modified Sequential Organ Failure Assessment score; CRP, C-reactive protein; WCC, White cell count; ACCP/ SCCM, American College of Chest Physicians/Society of Critical Care Medicine; SIRS, Systemic inflammatory response syndrome; SSC, Surviving Sepsis Campaign; HC, hydrocortisone; DX, dexamethasone; CI, Cardiac index; PAOP, pulmonary artery occlusion pressure; MODS, multiorgan dysfunction syndrome; MP, methylprednisolone; AA, Ascorbic acid; THI, thiamine

HC, DX, MP, AA, THI were given in IV if not otherwise specified.

FLU was given in via nasogastric route.

Table 4. Results of individual studies on short-term mortality (<90 days)

|  |  |  |  | Experimental | | Control | |  |
| --- | --- | --- | --- | --- | --- | --- | --- | --- |
| Study | Experimental | Control | Outcome | Events | Total | Events | Total | RR (95% CI) |
| Sprung 1984 | GLU | Placebo | Hospital | 33 | 43 | 11 | 16 | 1.12 [0.77; 1.61] |
| Bone 1987 | GLU | Placebo | 14-day | 65 | 191 | 48 | 190 | 1.35 [0.98; 1.84] |
| Luce 1987 | GLU | Placebo | Hospital | 22 | 38 | 20 | 37 | 1.07 [0.72; 1.60] |
| Hinshaw 1987 | GLU | Placebo | 14-day | 23 | 112 | 24 | 111 | 0.95 [0.57; 1.58] |
| Bollaert 1998 | GLU | Placebo | 28-day | 7 | 22 | 12 | 19 | 0.50 [0.25; 1.02] |
| Briegel 1999 | GLU | Placebo | ICU | 4 | 20 | 6 | 20 | 0.67 [0.22; 2.01] |
| Annane 2002 | GLU + FLU | Placebo | 28-day | 82 | 150 | 91 | 149 | 0.90 [0.74; 1.09] |
| Oppert 2005 | GLU | Placebo | 28-day | 7 | 18 | 11 | 23 | 0.81 [0.40; 1.67] |
| Cicarelli 2007 | GLU | Placebo | 28-day | 7 | 14 | 12 | 15 | 0.62 [0.35; 1.12] |
| Sprung 2008 | GLU | Placebo | 28-day | 86 | 251 | 78 | 248 | 1.09 [0.85; 1.40] |
| Meduri 2009 | GLU | Placebo | Hospital | 23 | 48 | 6 | 32 | 2.56 [1.17; 5.57] |
| Annane 2010 | GLU + FLU | GLU | Hospital | 105 | 245 | 121 | 264 | 0.94 [0.77; 1.14] |
| Gordon 2014 | GLU | Placebo | 28-day | 7 | 31 | 7 | 30 | 0.97 [0.39; 2.43] |
| Donnino 2016 | THI | Placebo | Hospital | 19 | 43 | 18 | 45 | 1.10 [0.68; 1.80] |
| Zabet 2016 | AA | Placebo | 28-day | 2 | 14 | 9 | 14 | 0.22 [0.06; 0.85] |
| Gordon 2016 | GLU | Placebo | 28-day | 56 | 201 | 53 | 207 | 1.09 [0.79; 1.50] |
| Tongyoo 2016 | GLU | Placebo | 28-day | 22 | 98 | 27 | 99 | 0.82 [0.50; 1.34] |
| Lv 2017 | GLU | Placebo | 28-day | 23 | 58 | 19 | 60 | 1.25 [0.77; 2.04] |
| Annane 2018 | GLU + FLU | Placebo | 28-day | 207 | 614 | 244 | 627 | 0.87 [0.75; 1.00] |
| Venkatesh 2018 | GLU | Placebo | 28-day | 410 | 1841 | 448 | 1840 | 0.91 [0.81; 1.03] |
| Fowler 2019 | AA | Placebo | 28-day | 25 | 84 | 38 | 82 | 0.64 [0.43; 0.96] |
| Harun 2019 | THI | Placebo | ICU | 14 | 32 | 23 | 33 | 1.20 [0.66; 2.19] |
| Fujii 2020 | GLU + AA + THI | GLU | 28-day | 24 | 106 | 21 | 103 | 1.11 [0.66; 1.87] |
| Moskowitz 2020 | GLU + AA + THI | Placebo | Hospital | 35 | 101 | 29 | 99 | 1.18 [0.79; 1.78] |
| Iglesias 2020 | GLU + AA + THI | Placebo | ICU | 6 | 68 | 10 | 69 | 0.61 [0.23; 1.58] |
| Wani 2020 | GLU + AA + THI | Placebo | 30-day | 20 | 50 | 21 | 50 | 0.95 [0.59; 1.52] |
| Reddy 2020 | GLU + AA + THI | GLU | Hospital | 4 | 9 | 2 | 9 | 2.00 [0.48; 8.31] |
|  | GLU + AA | GLU |  | 2 | 9 | 2 | 9 | 1.00 [0.18; 5.63] |
| Mohamed 2020 | GLU + AA + THI | GLU | Hospital | 26 | 45 | 23 | 43 | 1.08 [0.74; 1.57] |
| Hwang 2020 | AA + THI | Placebo | 28-day | 11 | 53 | 9 | 58 | 1.34 [0.60; 2.97] |
| Petsakul 2020 | THI | Placebo | 28-day | 5 | 25 | 7 | 25 | 0.71 [0.26; 1.95] |
| Chang 2020 | GLU + AA + THI | Placebo | 28-day | 11 | 40 | 14 | 40 | 0.79 [0.41; 1.52] |
| Aisa-Alvarez 2020 | AA vs. Placebo | Placebo | 28-day | 3 | 18 | 5 | 21 | 0.70 [0.19; 2.53] |
| Sevransky 2021 | GLU + AA + THI | Placebo | 30-day | 56 | 252 | 60 | 249 | 0.92 [0.67; 1.27] |

GLU, Glucocorticoid; AA, ascorbic acid; THI, Thiamine; FLU, Fludrocortisone; ICU, Intensive Care Unit

Table 5. Results of individual studies on longer-term mortality (≥90 days)

|  |  |  |  | Experimental | | Control | |  |
| --- | --- | --- | --- | --- | --- | --- | --- | --- |
| Study | Experimental | Control | Outcome | Events | Total | Events | Total | RR (95% CI) |
| Briegel 1999 | GLU | Placebo | 1-year | 15 | 20 | 14 | 20 | 1.07 [0.73;1.57] |
| Annane 2002 | GLU + FLU | Placebo | 1-year | 102 | 150 | 112 | 149 | 0.90 [0.78; 1.04] |
| Sprung 2008 | GLU | Placebo | 1-year | 137 | 242 | 127 | 235 | 1.05 [0.89; 1.23] |
| Annane 2018 | GLU + FLU | Placebo | 180-day | 285 | 611 | 328 | 625 | 0.89 [0.79; 1.00] |
| Venkatesh 2018 | GLU | Placebo | 90-day | 511 | 1832 | 526 | 1826 | 0.97 [0.87; 1.07] |
| Hwang 2020 | AA + THI | Placebo | 90-day | 17 | 53 | 16 | 58 | 1.16 [0.66; 2.66] |
| Sevransky 2021 | GLU + AA + THI | Placebo | 180-day | 101 | 252 | 94 | 249 | 1.07 [0.86; 1.33] |

GLU, Glucocorticoid; AA, ascorbic acid; THI, Thiamine; FLU, Fludrocortisone;

Table 6. Results of individual studies on time to resolution of shock

|  |  |  | Experimental | | | Control | | |  |
| --- | --- | --- | --- | --- | --- | --- | --- | --- | --- |
| Study | Experimental | Control | n | Mean | SD | n | Mean | SD | MD (95% CI) |
| Briegel 1999 | GLU | Placebo | 20 | 3.00 | 3.99 | 20 | 9.67 | 12.77 | -6.67  [-12.53; -0.81] |
| Oppert 2005 | GLU | Placebo | 18 | 2.56 | 2.04 | 23 | 5.07 | 4.91 | -2.51  [-4.73; -0.30] |
| Cicarelli 2007 | GLU | Placebo | 14 | 3.00 | 1.18 | 15 | 3.80 | 0.76 | -0.80  [-1.53; -0.07] |
| Zabet 2016 | AA | Placebo | 14 | 2.07 | 1.07 | 14 | 2.98 | 0.07 | -0.91  [-1.48; -0.35] |
| Gordon 2016 | GLU | Placebo | 202 | 2.18 | 1.71 | 207 | 2.35 | 2.27 | -0.17  [-0.56; 0.22] |
| Tongyoo 2016 | GLU | Placebo | 98 | 4.80 | 3.00 | 99 | 6.80 | 5.70 | -2.00  [-3.27; -0.73] |
| Lv 2017 | GLU | Placebo | 58 | 3.50 | 2.40 | 60 | 3.80 | 4.00 | -0.30  [-1.49; 0.89] |
| Venkatesh 2018 | GLU | Placebo | 1853 | 3.33 | 2.23 | 1860 | 5.00 | 5.19 | -1.67  [-1.92; -1.41] |
| Harun 2019 | THI | Placebo | 32 | 3.54 | 2.69 | 33 | 3.93 | 3.20 | -0.39  [-1.83; 1.04] |
| Iglesias 2020 | GLU + AA + THI | Placebo | 68 | 1.12 | 0.92 | 69 | 2.21 | 1.58 | -1.08  [-1.52; -0.65] |
| Wani 2020 | GLU + AA + THI | Placebo | 50 | 3.15 | 1.226 | 50 | 4.01 | 1.69 | -0.85  [-1.43; -0.27] |
| Reddy 2020 | GLU + AA + THI | GLU | 5 | 1.29 | 0.52 | 5 | 5.15 | 5.80 | -3.86  [-8.96; 1.24] |
|  | GLU + AA | GLU | 7 | 1.75 | 2.14 | 5 | 5.15 | 5.80 | -3.40  [-8.72; 1.92] |
| Mohamed 2020 | GLU + AA + THI | GLU | 45 | 1.44 | 0.94 | 43 | 1.89 | 1.02 | -0.45  [-0.86; -0.04] |
| Chang 2020 | GLU + AA + THI | Placebo | 40 | 2.39 | 2.52 | 40 | 2.65 | 2.43 | -0.25  [-1.34; 0.83] |

GLU, Glucocorticoid; AA, ascorbic acid; THI, Thiamine; FLU, Fludrocortisone;

Table 7. Results of individual studies on duration of mechanical ventilation

|  |  |  | Experimental | | | Control | | |  |
| --- | --- | --- | --- | --- | --- | --- | --- | --- | --- |
| Study | Experimental | Control | n | Mean | SD | n | Mean | SD | MD (95% CI) |
| Cicarelli 2007 | GLU | Placebo | 14 | 3.40 | 2.50 | 15 | 4.00 | 3.20 | -0.60  [-2.68; 1.48] |
| Gordon 2016 | GLU | Placebo | 202 | 6.33 | 7.47 | 207 | 7.00 | 6.71 | -0.67  [-2.04; 0.71] |
| Tongyoo 2016 | GLU | Placebo | 98 | 11.80 | 7.80 | 99 | 13.90 | 9.00 | -2.10  [-4.45; 0.25] |
| Venkatesh 2018 | GLU | Placebo | 1853 | 9.00 | 11.13 | 1860 | 11.33 | 15.60 | -2.33  [-3.20; -1.46] |
| Hwang 2020 | AA + THI | Placebo | 23 | 7.00 | 7.11 | 24 | 6.00 | 3.94 | 1.00  [-2.31; 4.31] |
| Chang 2020 | GLU + AA + THI | Placebo | 40 | 5.96 | 5.63 | 40 | 4.80 | 5.49 | 1.17  [-1.27; 3.60] |

GLU, Glucocorticoid; AA, ascorbic acid; THI, Thiamine; FLU, Fludrocortisone;

Table 8. Results of individual studies on ICU length of stay

|  |  |  | Experimental | | | Control | | |  |
| --- | --- | --- | --- | --- | --- | --- | --- | --- | --- |
| Study | Experimental | Control | n | Mean | SD | n | Mean | SD | MD (95% CI) |
| Sprung 2008 | GLU | Placebo | 251 | 19.00 | 31.00 | 248 | 18.00 | 17.00 | 1.00 [-3.38; 5.38] |
| Annane 2010 | GLU + FLU | GLU | 245 | 9.67 | 8.95 | 264 | 10.17 | 10.06 | -0.50 [-2.15; 1.15] |
| Donnino 2016 | THI | Placebo | 43 | 8.33 | 6.90 | 45 | 9.33 | 11.49 | -1.00 [-4.94; 2.94] |
| Zabet 2016 | AA | Placebo | 14 | 21.45 | 20.57 | 14 | 10.23 | 13.04 | 11.22 [-1.54; 23.98] |
| Gordon 2016 | GLU | Placebo | 202 | 6.67 | 5.97 | 207 | 7.00 | 6.72 | -0.33 [-1.56; 0.90] |
| Lv 2017 | GLU | Placebo | 58 | 10.90 | 17.50 | 60 | 10.20 | 13.10 | 0.70 [-4.89; 6.29] |
| Venkatesh 2018 | GLU | Placebo | 1853 | 15.0 | 18.54 | 1860 | 20.00 | 26.71 | -5.00 [-6.48; -3.52] |
| Harun 2019 | THI | Placebo | 32 | 6.67 | 5.43 | 33 | 7.00 | 6.97 | -0.33 [-3.37; 2.70] |
| Mohamed 2020 | GLU + AA + THI | GLU | 45 | 12.44 | 14.20 | 43 | 8.44 | 8.16 | 4.00 [-0.81; 8.81] |
| Hwang 2020 | AA + THI | Placebo | 45 | 6.33 | 6.12 | 52 | 7.33 | 6.48 | -1.00 [-3.50; 1.50] |

GLU, Glucocorticoid; AA, ascorbic acid; THI, Thiamine; FLU, Fludrocortisone;

Table 9. Results of individual studies on hospital length of stay

|  |  |  | Experimental | | | Control | | |  |
| --- | --- | --- | --- | --- | --- | --- | --- | --- | --- |
| Study | Experimental | Control | n | Mean | SD | n | Mean | SD | MD (95% CI) |
| Sprung 2008 | GLU | Placebo | 251 | 34.00 | 41.00 | 248 | 34.00 | 37.00 | 0 [-6.85; 6.85] |
| Annane 2010 | GLU + FLU | GLU | 245 | 15.00 | 14.17 | 264 | 19.67 | 20.13 | -4.67 [-7.67; -1.66] |
| Donnino 2016 | THI | Placebo | 43 | 13.67 | 9.20 | 45 | 14.67 | 13.01 | -1.00 [-5.69; 3.69] |
| Gordon 2016 | GLU | Placebo | 202 | 18.67 | 20.16 | 207 | 21.00 | 23.14 | -2.33 [-6.54; 1.87] |
| Lv 2017 | GLU | Placebo | 58 | 23.70 | 36.80 | 60 | 21.70 | 21.70 | 2.00 [-8.95; 12.95] |
| Fujii 2020 | GLU + AA + THI | GLU | 107 | 14.83 | 14.88 | 103 | 14.87 | 14.96 | -0.03 [-4.07; 4.00] |
| Wani 2020 | GLU + AA + THI | Placebo | 50 | 11.82 | 7.36 | 50 | 10.70 | 6.39 | 1.12 [-1.58; 3.82] |
| Mohamed 2020 | GLU + AA + THI | GLU | 45 | 31.58 | 31.06 | 43 | 20.90 | 15.01 | 10.68 [0.56; 20.80] |
| Hwang 2020 | AA + THI | Placebo | 53 | 15.33 | 7.62 | 58 | 16.17 | 12.92 | -0.83 [-4.74; 3.07] |

GLU, Glucocorticoid; AA, ascorbic acid; THI, Thiamine; FLU, Fludrocortisone;

Table 10. Results of individual studies on secondary infections

|  |  |  | Experimental | | Control | |  |
| --- | --- | --- | --- | --- | --- | --- | --- |
| Study | Experimental | Control | Events | Total | Events | Total | RR (95% CI) |
| Sprung 1984 | GLU | Placebo | 11 | 43 | 1 | 16 | 4.09 [0.57; 29.20] |
| Bone 1987 | GLU | Placebo | 29 | 152 | 30 | 147 | 0.93 [0.59; 1.48] |
| Luce 1987 | GLU | Placebo | 3 | 37 | 4 | 36 | 0.73 [0.18; 3.03] |
| Hinshaw 1987 | GLU | Placebo | 16 | 112 | 23 | 111 | 0.69 [0.39; 1.23] |
| Bollaert 1998 | GLU | Placebo | 7 | 22 | 9 | 19 | 0.67 [0.31; 1.46] |
| Briegel 1999 | GLU | Placebo | 10 | 20 | 7 | 20 | 1.43 [0.68; 3.00] |
| Annane 2002 | GLU + FLU | Placebo | 22 | 150 | 27 | 149 | 0.81 [0.48; 1.35] |
| Cicarelli 2007 | GLU | Placebo | 0 | 14 | 1 | 15 | 0.36 [0.02; 8.07] |
| Sprung 2008 | GLU | Placebo | 78 | 234 | 61 | 232 | 1.27 [0.96; 1.68] |
| Annane 2010 | GLU + FLU | GLU | 53 | 245 | 37 | 264 | 1.54 [1.05; 2.26] |
| Tongyoo 2016 | GLU | Placebo | 34 | 98 | 41 | 99 | 0.84 [0.59; 1.20] |
| Annane 2018 | GLU + FLU | Placebo | 191 | 614 | 176 | 626 | 1.11 [0.93; 1.31] |
| Moskowitz 2020 | GLU + AA + THI | Placebo | 13 | 101 | 12 | 99 | 1.06 [0.51; 2.21] |
| Chang 2020 | GLU + AA + THI | Placebo | 1 | 40 | 0 | 40 | 3.00 [0.13; 71.48] |

GLU, Glucocorticoid; AA, ascorbic acid; THI, Thiamine; FLU, Fludrocortisone;

Table 11. Results of individual studies on gastrointestinal bleeding

|  |  |  | Experimental | | Control | |  |
| --- | --- | --- | --- | --- | --- | --- | --- |
| Study | Experimental | Control | Events | Total | Events | Total | RR (95% CI) |
| Sprung 1984 | GLU | Placebo | 1 | 43 | 2 | 16 | 0.19 [0.02; 1.91] |
| Luce 1987 | GLU | Placebo | 18 | 37 | 16 | 36 | 1.09 [0.67; 1.79] |
| Hinshaw 1987 | GLU | Placebo | 14 | 112 | 10 | 111 | 1.39 [0.64; 2.99] |
| Bollaert 1998 | GLU | Placebo | 1 | 22 | 3 | 19 | 0.29 [0.03; 2.54] |
| Briegel 1999 | GLU | Placebo | 1 | 20 | 0 | 20 | 3.00 [0.13; 69.42] |
| Annane 2002 | GLU + FLU | Placebo | 11 | 150 | 8 | 149 | 1.37 [0.57; 3.30] |
| Sprung 2008 | GLU | Placebo | 15 | 234 | 13 | 232 | 1.14 [0.56; 2.35] |
| Tongyoo 2016 | GLU | Placebo | 3 | 98 | 4 | 99 | 0.76 [0.17; 3.30] |
| Annane 2018 | GLU + FLU | Placebo | 39 | 614 | 45 | 626 | 0.88 [0.58; 1.34] |
| Fujii 2020 | GLU + AA + THI | GLU | 0 | 106 | 1 | 103 | 0.32 [0.01; 7.86] |
| Chang 2020 | GLU + AA + THI | Placebo | 3 | 40 | 2 | 40 | 1.50 [0.26; 8.50] |

GLU, Glucocorticoid; AA, ascorbic acid; THI, Thiamine; FLU, Fludrocortisone;

Table 12. Results of individual studies on delirium

|  |  |  | Experimental | | Control | |  |
| --- | --- | --- | --- | --- | --- | --- | --- |
| Study | Experimental | Control | Events | Total | Events | Total | RR (95% CI) |
| Hinshaw 1987 | GLU | Placebo | 9 | 112 | 8 | 111 | 1.11 [0.45; 2.79] |
| Annane 2002 | GLU + FLU | Placebo | 0 | 150 | 1 | 149 | 0.33 [0.01; 8.06] |
| Sprung 2008 | GLU | Placebo | 1 | 234 | 1 | 232 | 0.99 [0.06; 15.76] |
| Venkatesh 2018 | GLU | Placebo | 3 | 1835 | 0 | 1829 | 6.98 [0.36; 134.98] |

GLU, Glucocorticoid; AA, ascorbic acid; THI, Thiamine; FLU, Fludrocortisone;

Table 13. Results of individual studies on hyperglycemia

|  |  |  |  | Experimental | | Control | |  |
| --- | --- | --- | --- | --- | --- | --- | --- | --- |
| Study | Experimental | Control | Outcome | Events | Total | Events | Total | RR (95% CI) |
| Sprung 1984 | GLU | Placebo | >200mg/dL | 4 | 32 | 0 | 16 | 3.41 [0.19; 59.98] |
| Luce 1987 | GLU | Placebo | >250mg/dL | 16 | 37 | 15 | 36 | 1.04 [0.61; 1.77] |
| Hinshaw 1987 | GLU | Placebo | NR | 17 | 112 | 23 | 111 | 0.73 [0.41; 1.29] |
| Sprung 2008 | GLU | Placebo | >150mg/dL | 186 | 234 | 161 | 232 | 1.15 [1.03; 1.28] |
| Tongyoo 2016 | GLU | Placebo | >150mg/dL | 79 | 98 | 67 | 99 | 1.19 [1.91; 1.41] |
| Annane 2018 | GLU + FLU | Placebo | ≥150mg/dL | 547 | 614 | 520 | 626 | 1.07 [1.03; 1.12] |
| Venkatesh 2018 | GLU | Placebo | NR | 6 | 1835 | 3 | 1829 | 1.99 [0.50; 7.96] |
| Fujii 2020 | GLU + AA + THI | GLU | NR | 1 | 106 | 0 | 103 | 2.92 [0.12; 70.75] |
| Moskowitz 2020 | GLU + AA + THI | Placebo | >300mg/dL | 12 | 101 | 7 | 99 | 1.68 [0.69; 4.09] |

GLU, Glucocorticoid; AA, ascorbic acid; THI, Thiamine; FLU, Fludrocortisone; NR, not reported

Table 14. Results of individual studies on hypernatremia

|  |  |  |  | Experimental | | Control | |  |
| --- | --- | --- | --- | --- | --- | --- | --- | --- |
| Study | Experimental | Control | Outcome | Events | Total | Events | Total | RR (95% CI) |
| Hinshaw 1987 | GLU | Placebo | NR | 9 | 112 | 15 | 111 | 0.59 [0.27; 1.30] |
| Sprung 2008 | GLU | Placebo | >150mmol/L | 67 | 234 | 42 | 232 | 1.58 [1.13; 2.22] |
| Venkatesh 2018 | GLU | Placebo | NR | 3 | 1835 | 0 | 1829 | 6.98 [0.36; 134.98] |
| Moskowitz 2020 | GLU + AA + THI | Placebo | >150mmol/L | 11 | 101 | 7 | 99 | 1.54 [0.62; 3.81] |
| Chang 2020 | GLU + AA + THI | Placebo | >160mmol/L | 13 | 40 | 3 | 40 | 4.33 [1.34; 14.05] |

GLU, Glucocorticoid; AA, ascorbic acid; THI, Thiamine; FLU, Fludrocortisone; NR, not reported

Table 15. Investigation of transitivity assumption

| Study information | | Intervention | Patient characteristics | | |
| --- | --- | --- | --- | --- | --- |
| Comparison | Study ID | Dosage of glucocorticoid  (on the first day) | Age, mean (SD)^a^ | APACHE II, mean (SD)^a^ | Vasopressor/ Inotrope dependence at baseline (%) |
| GLU vs. Placebo | Sprung 1984 | MP 30-60 mg/kg  Or DX 6-12mg/kg | 54.0 (2.0) | NA | 93.2 |
| GLU vs. Placebo | Bone 1987 | MP 120 mg/kg | 53.6 (16.0) | NA | NA |
| GLU vs. Placebo | Luce 1987 | MP 120 mg/kg | 51.5 (2.9) | NA | 44.0 |
| GLU vs. Placebo | Hinshaw 1987 | MP 75mg/kg | 60.8 (NA) | NA | NA |
| GLU vs. Placebo | Bollaert 1998 | HC 300mg | 59.1 (15.6) | NA | 100 |
| GLU vs. Placebo | Briegel 1999 | HC 100mg + 4.32mg/kg | 49.0 (4.4) | 26.5 (1.1) | 100 |
| GLU + FLU vs. Placebo | Annane 2002 | HC 200mg | 61.0 (16.0) | NA | 93.3 |
| GLU vs. Placebo | Oppert 2005 | HC 50mg + 4.32mg/kg | 52.3 (NA) | 24.7 (7.9) | 100 |
| GLU vs. Placebo | Cicarelli 2007 | DX 0.4mg/kg | 64.0 (13.0) | 19.5 (4.5) | NA |
| GLU vs. Placebo | Sprung 2008 | HC 200mg | 63.0 (14.5) | NA | 98.6 |
| GLU vs. Placebo | Meduri 2009 | HC 240mg | NA | NA | NA |
| GLU + FLU vs. GLU | Annane 2010 | HC 200mg | 63.9 (2.68) | NA | 100 |
| GLU vs. Placebo | Gordon 2014 | HC 200mg | 61.2 (17.0) | 19.5 (6.3) | 100 |
| GLU vs. Placebo | Mirea 2014 | HC 200mg | NA | NA | NA |
| THI vs. Placebo | Donnino 2016 | - | 67.4 (15.7) | 26.1 (9.1) | 100 |
| AA vs. Placebo | Zabet 2016 | - | 63.9 (12.8) | 21.0 (5.7) | 100 |
| GLU vs. Placebo | Gordon 2016 | HC 200mg | 65.7 (17.1) | 24.3 (8.2) | 100 |
| GLU vs. Placebo | Tongyoo 2016 | HC 200mg | 64.4 (16.6) | 21.7 (5.7) | 78.2 |
| GLU vs. Placebo | Lv 2017 | HC 200mg | 66.8 (14.9) | 23.4 (8.5) | 100 |
| GLU + FLU vs. Placebo | Annane 2018 | HC 200mg | 66.0 (14.5) | NA | 100 |
| GLU vs. Placebo | Venkatesh 2018 | HC 200mg | 62.5 (15.1) | 23.7 (7.8) | 99.6 |
| AA vs. Placebo | Fowler 2019 | - | 55.2 (16.6) | NA | 56.5 |
| THI vs. Placebo | Harun 2019 | - | 64.0 (16.4) | 31.1 | NA |
| GLU + AA + THI vs. GLU | Fujii 2020 | HC 200mg | 61.8 (14.9) | NA | 100 |
| GLU + AA + THI vs. Placebo | Moskowitz 2020 | HC 200mg | 68.3 (14.4) | NA | 100 |
| GLU + AA + THI vs. Placebo | Iglesias 2020 | HC 200mg | 68.5 (13.1) | 24.5 (8.2) | 75.2 |
| GLU + AA + THI vs. Placebo | Wani 2020 | HC 200mg | 51.5 (35.7) | 19.5 (7.1) | 84.0 |
| GLU + AA + THI vs. GLU + AA  vs. GLU | Reddy 2020 | HC 200mg | 55.2 (11.4) | 18.9 (4.7) | 100 |
| GLU + AA + THI vs. Placebo | Mohamed 2020 | HC 200mg | 59.0 (14.9) | NA | 100 |
| AA + THI vs. Placebo | Hwang 2020 | - | 68.8 (9.9) | 23.2 (12.5) | 100 |
| THI vs. Placebo | Petsakul 2020 | - | 65.5 (17.8) | 27.5 (7.0) | 100 |
| GLU + AA + THI vs. Placebo | Chang 2020 | HC 200mg | 61.6 (14.0) | 23.0 (8.0) | 100 |
| AA vs. Placebo | Aisa-Alvarez 2020 | - | 67.1 (16.6) | NA | NA |
| GLU + AA + THI vs. Placebo | Sevransky 2021 | HC 200mg | 60.8 (15.0) | 26.8 (9.4) | 38 |

GLU, Glucocorticoid; AA, ascorbic acid; THI, Thiamine; FLU, Fludrocortisone;
HC, hydrocortisone; MP, methylprednisolone; DX, dexamethasone

^a^Where data was not available, the median and interquartile range were converted into mean and standard deviation using a published equation, and combined mean and standard deviation were calculated using the Cochrane formula. (1, 2)

Table 16. Direct, indirect, and network meta-analysis estimates of glucocorticoid, ascorbic acid, and thiamine in patients with sepsis and septic shock on short term mortality

| Comparison | No. of trials | Direct estimates (RR; 95% CI) | Quality | Indirect estimates  (RR; 95% CI) | Quality | Network estimates^a^  (RR; 95% CI) | Quality | P-value |
| --- | --- | --- | --- | --- | --- | --- | --- | --- |
| AA vs. AA + THI | 0 | - | - | 0.45 [0.19; 1.08] | Moderate^b^ | 0.45 [0.19; 1.08] | Moderate | - |
| AA vs. GLU | 0 | - | - | 0.61 [0.42; 0.89] | Low^c^ | 0.61 [0.42; 0.89] | Low | - |
| AA vs. GLU + AA | 0 | - | - | 0.58 [0.35; 0.97] | Very low^b,c^ | 0.91 [0.28; 2.91] | Very low | - |
| AA vs. GLU + AA+ THI | 0 | - | - | 0.59 [0.40; 0.89] | Moderate^d^ | 0.59 [0.40; 0.89] | Moderate | - |
| AA vs. GLU + FLU | 0 | - | - | 0.67 [0.46; 0.99] | Moderate^d^ | 0.67 [0.46; 0.99] | Moderate | - |
| AA vs. Placebo | 3 | 0.60 [0.41; 0.86] | Moderate^e^ | - | - | 0.60 [0.41; 0.86] | Moderate | - |
| AA vs. THI | 0 | - | - | 0.55 [0.33; 0.92] | Low^c^ | 0.55 [0.33; 0.92] | Low^c^ | - |
| AA + THI vs. GLU | 0 | - | - | 1.37 [0.62; 3.06] | Moderate^b^ | 1.37 [0.62; 3.06] | Moderate | - |
| AA + THI vs. GLU + AA | 0 | - | - | 2.03 [0.52;7.94] | Very low^b,c^ | 2.03 [0.52;7.94] | Very low | - |
| AA + THI vs. GLU + AA + THI | 0 | - | - | 1.33 [0.59; 3.01] | Low^b,d^ | 1.33 [0.59; 3.01] | Low | - |
| AA + THI vs. GLU + FLU | 0 | - | - | 1.51 [0.68; 3.38] | Moderate^b^ | 1.51 [0.68; 3.38] | Moderate | - |
| AA + THI vs. Placebo | 1 | 1.34 [0.60; 2.97] | Moderate^b^ | - | - | 1.34 [0.60; 2.97] | Moderate | - |
| AA + THI vs. THI | 0 | - | - | 1.24 [0.52; 2.97] | Very low^b,c^ | 1.24 [0.52; 2.97] | Very low | - |
| GLU vs. GLU + AA | 1 | 1.00 [0.18; 5.64] | Very low^f,g^ | 1.95 [0.46; 8.18] | Very low^c,f^ | 1.48 [0.49; 4.48] | Very low | 0.561 |
| GLU vs. GLU + AA + THI | 2 | 0.84 [0.51; 1.37] | Low^b,e^ | 0.99 [0.82; 1.21] | Low^b,d^ | 0.97 [0.81; 1.16] | Low | 0.536 |
| GLU vs. GLU + FLU | 1 | 1.07 [0.87; 1.31] | Moderate^b^ | 1.12 [0.97; 1.29] | Moderate^b^ | 1.10 [0.98; 1.24] | Moderate | 0.720 |
| GLU vs. Placebo | 15 | 0.99 [0.91; 1.08] | Moderate^b^ | 0.91 [0.74; 1.13] | Low^b,d^ | 0.98 [0.90; 1.05] | Low | 0.513 |
| GLU vs. THI | 0 | - | - | 0.90 [0.63; 1.30] | Very low^c,f^ | 0.90 [0.63; 1.30] | Very low | - |
| GLU + AA vs. GLU + AA+ THI | 1 | 0.50 [0.12; 2.08] | Very low^b,g^ | 0.97 [0.17; 5.54] | Very low^b,c^ | 0.65 [0.22; 1.97] | Very low | 0.561 |
| GLU + AA vs. GLU + FLU | 0 | - | - | 0.74 [0.24; 2.25] | Very low^b,c^ | 0.74 [0.24; 2.25] | Very low | - |
| GLU + AA vs. Placebo | 0 | - | - | 0.66 [0.22; 1.98] | Very low^b,c^ | 0.66 [0.22; 1.98] | Very low | - |
| GLU + AA vs. THI | 0 | - | - | 0.61 [0.19; 1.94] | Very low^b,c^ | 0.61 [0.19; 1.94] | Very low | - |
| GLU + AA + THI vs. GLU + FLU | 0 | - | - | 1.14 [0.93; 1.38] | Moderate^b^ | 1.14 [0.93; 1.38] | Moderate | - |
| GLU + AA + THI vs. Placebo | 6 | 0.98 [0.82; 1.18] | Moderate^b^ | 1.19 [0.74; 1.93] | Moderate^b^ | 1.01 [0.85; 1.19] | Moderate | 0.460 |
| GLU + AA + THI vs. THI | 0 | - | - | 0.93 [0.63; 1.38] | Low^b,d^ | 0.93 [0.63; 1.38] | Low | - |
| GLU + FLU vs. Placebo | 2 | 0.88 [0.78; 0.99] | High | 0.92 [0.74; 1.13] | Moderate^b^ | 0.89 [0.80; 0.98] | High | 0.720 |
| GLU + FLU vs. THI | 0 | - | - | 0.82 [0.57; 1.19] | Low^b,d^ | 0.82 [0.57; 1.19] | Low | - |
| THI vs. Placebo | 3 | 1.08 [0.76; 1.54] | Low^b,e^ | - | - | 1.08 [0.76; 1.54] | Low | - |

GLU, Glucocorticoid; AA, ascorbic acid; THI, Thiamine; FLU, Fludrocortisone

^a^ from standard NMA

^b^Quality of evidence rated down by one level for serious imprecision

^c^Contributing direct evidence of low or very low quality

^d^Contributing direct evidence of Moderate quality

^e^Quality of evidence rated down by one level for risk of bias

^f^Quality of evidence rated down by two levels for very serious imprecision

^g^Quality of evidence rated down by two levels for serious risk of bias

Table 17. Inconsistency tests for short-term mortality

| Node splitting method |
| --- |
| 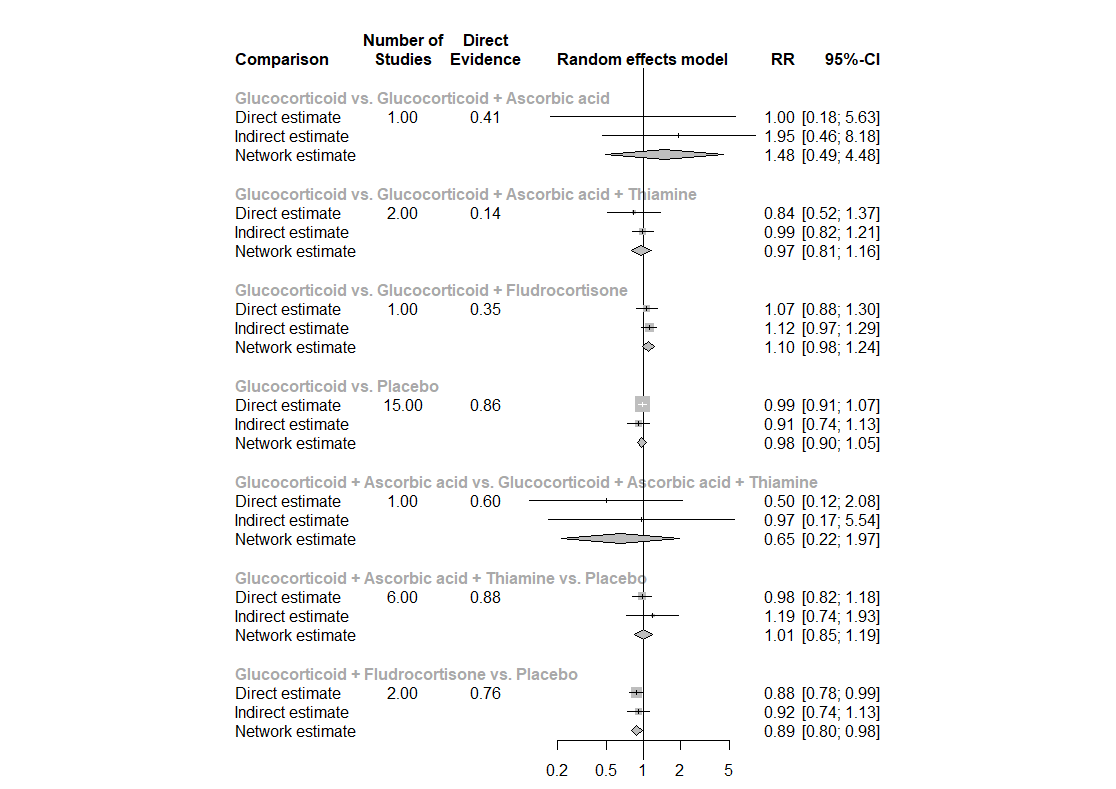 |
| Design by treatment |
| Q=1.03, df=3, p-value=0.794 |

Table 18. Adverse events reported by studies potentially related to ascorbic acid or thiamine

| Studies | Interventions | | Potential adverse events |
| --- | --- | --- | --- |
|  | Ascorbic acid | Thiamine |  |
| Zabet 2016 | X |  | No ascorbic acid related adverse events were identified |
| Harun 2019 | X | X | Nil reported |
| Fowler 2019 | X |  | No unexpected study-related adverse events occurred during the trial |
| Fujii 2020 | X | X | Nil reported |
| Moskowitz 2020 | X | X | No recorded serious allergic reactions  2 cases of renal calculus in intervention arm (n=101) and 0 cases in placebo arm (n=99) |
| Iglesias 2020 | X | X | No adverse events were noted that were deemed related to the study drug. |
| Wani 2020 | X | X | Nil reported |
| Reddy 2020 | X | X | Nil reported |
| Mohamed 2020 | X | X | No adverse events attributable to vitamin C |
| Hwang 2020 | X | X | No skin rash, anaphylaxis, gastrointestinal symptoms, or symptomatic ureter or renal stones in intervention group |
| Petsakul 2020 |  | X | No rash, itchy, red skin or anaphylaxis in intervention group |
| Chang 2020 | X | X | Nil reported |
| Aisa-Alvarez 2020 | X |  | 1 abdominal pain and 1 skin rash in intervention group |
| Sevransky 2021 | X | X | No reported serious adverse events  2 adverse events (haemorrhagic shock and worsening kidney function) in intervention group |

Figure 1. PRISMA flow diagram of the search results

Full-text articles excluded:

Non-RCT (n = 12)

Not in English (n=3)

Not patients with sepsis/ septic shock (n = 11)

Not related to steroid/ ascorbic acid/ thiamine (n=9)

Records excluded
(n = 2422)

Records after duplicates removed
(n = 2491)

Full-text articles assessed for eligibility
(n = 69)

Studies included in meta-analysis

(n = 34)

2563 potentially relevant citations identified

2553 through database

19 from other sources
(n = 890)

Figure 2. Summary of risk of bias assessment


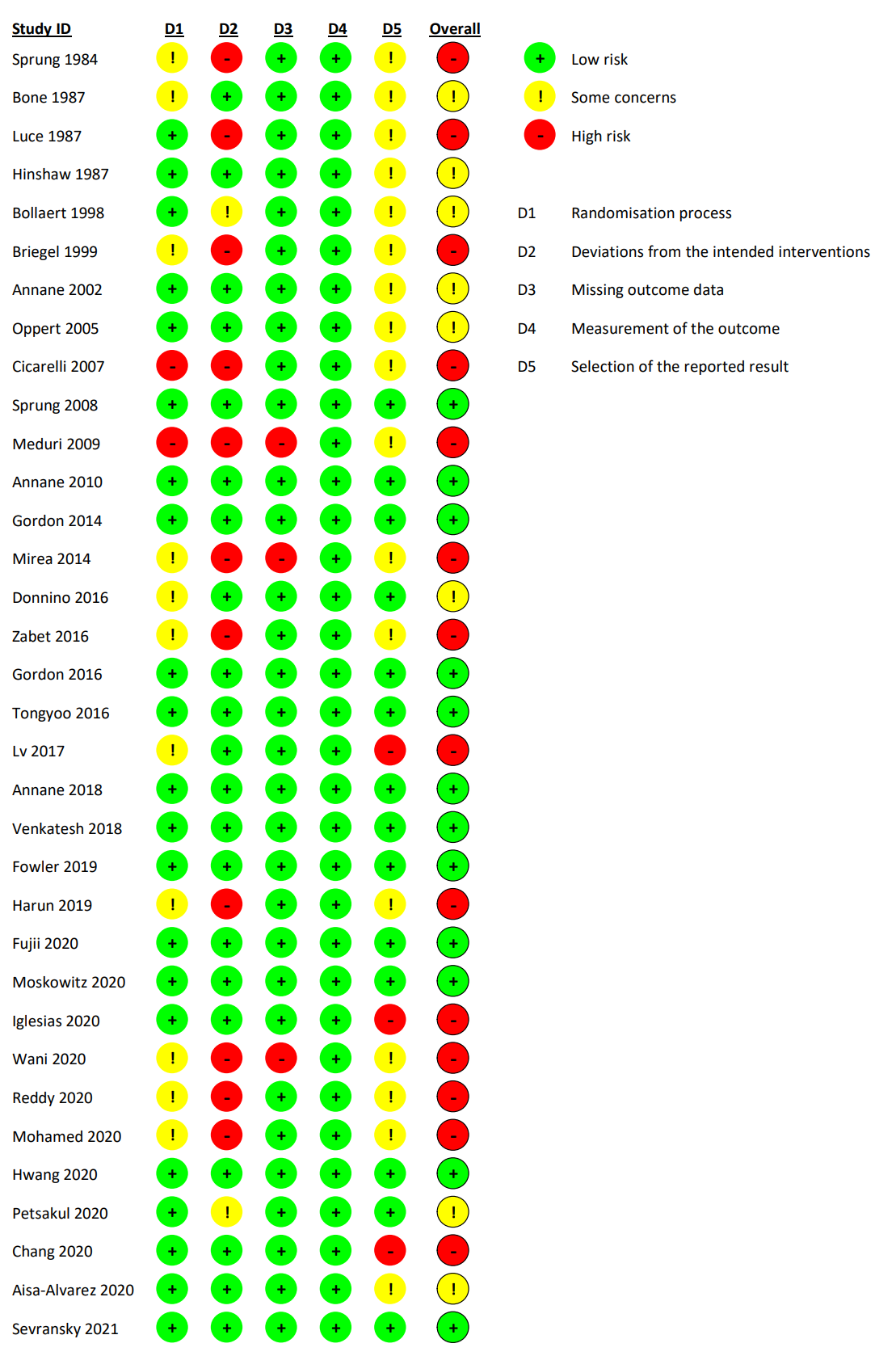


Figure 3. Network geometry of studies presenting short-term mortality (<90 days)


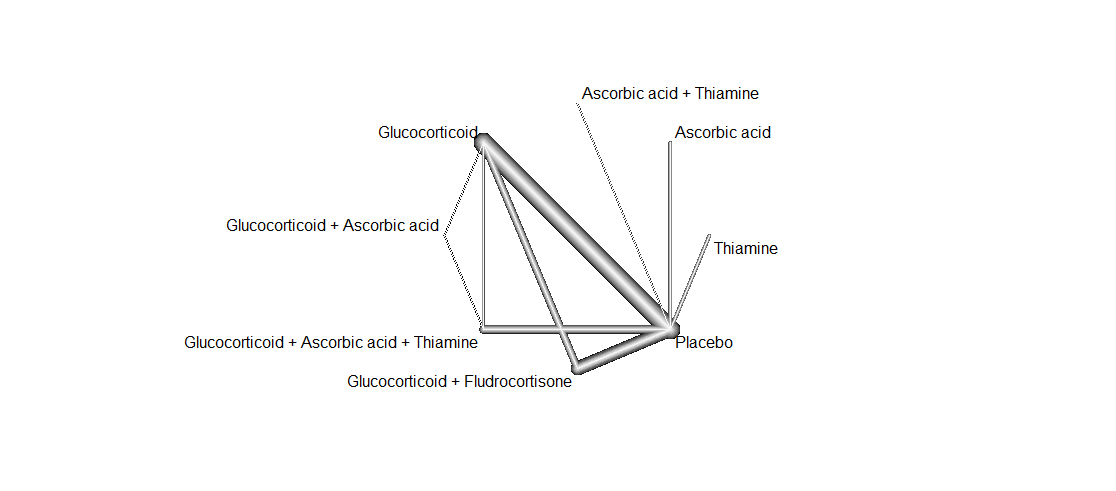


Figure 4. Forest plot for components on short-term mortality (<90 days)


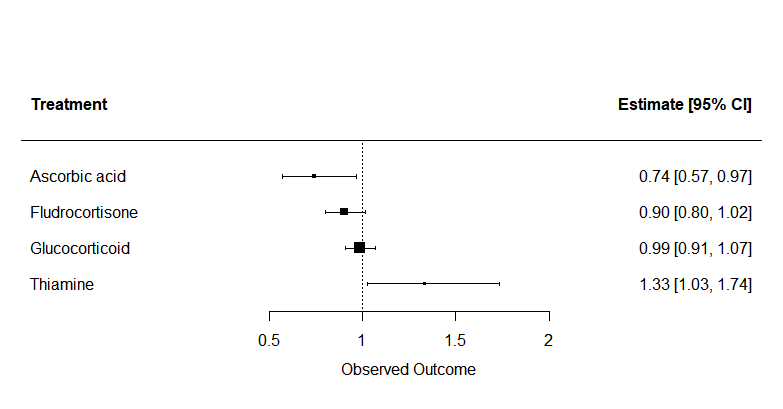
Estimate referred to risk ratio.

Figure 5. Funnel plot of network meta-analysis on short-term mortality (<90 days)


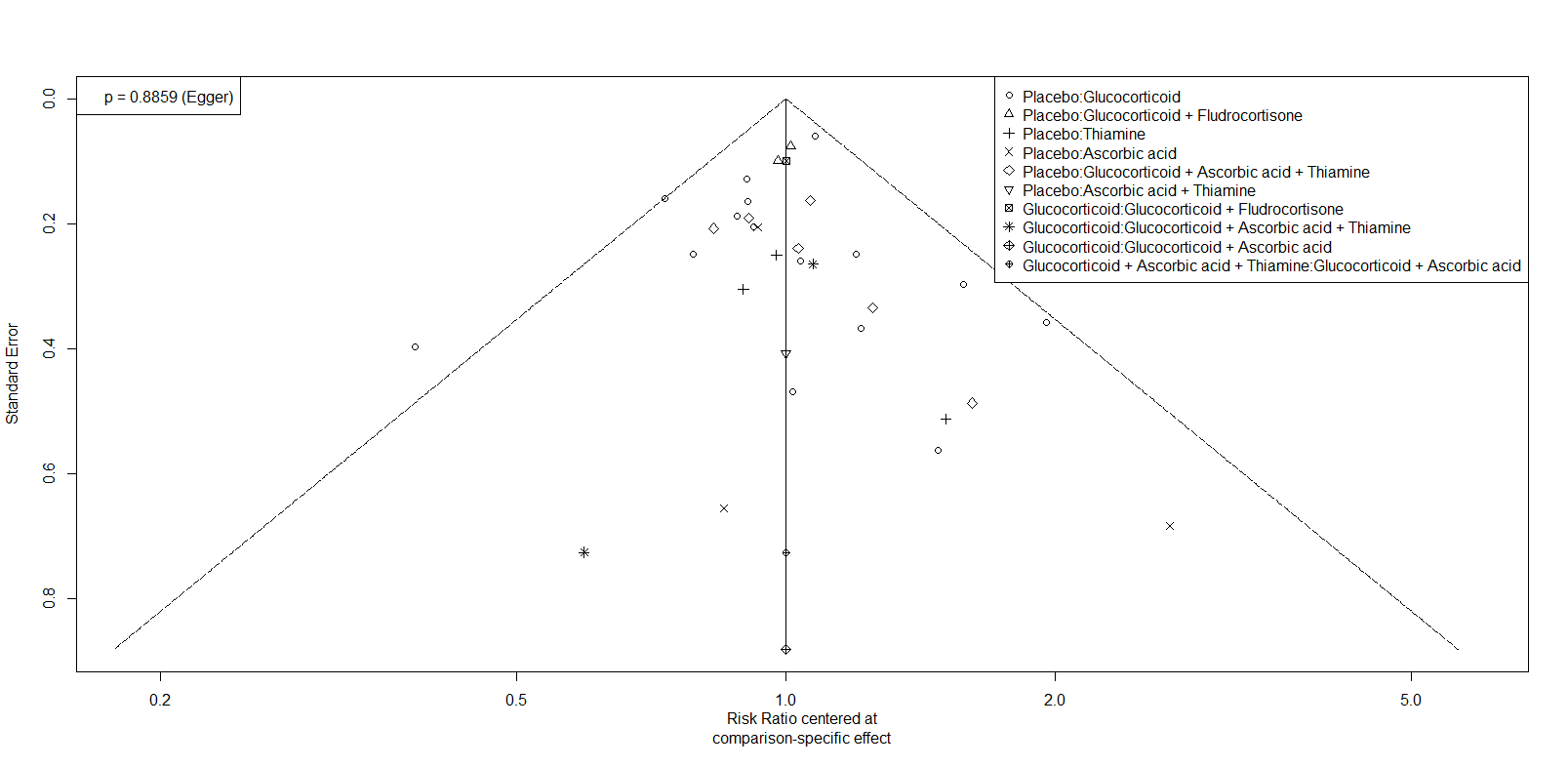


Figure 6. Network geometry of studies presenting longer-term mortality (≥ 90 days)


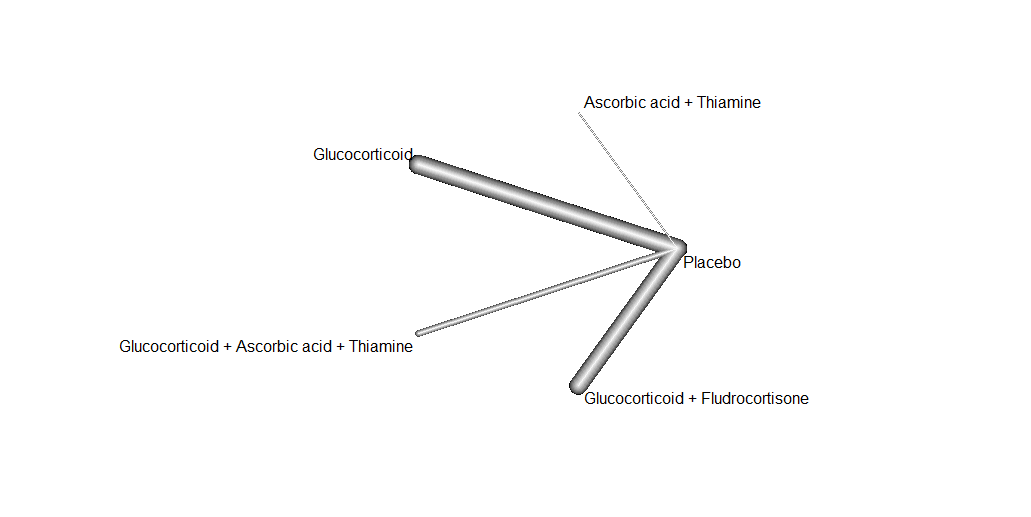


Figure 7. Network meta-analysis on longer-term mortality (≥ 90 days)


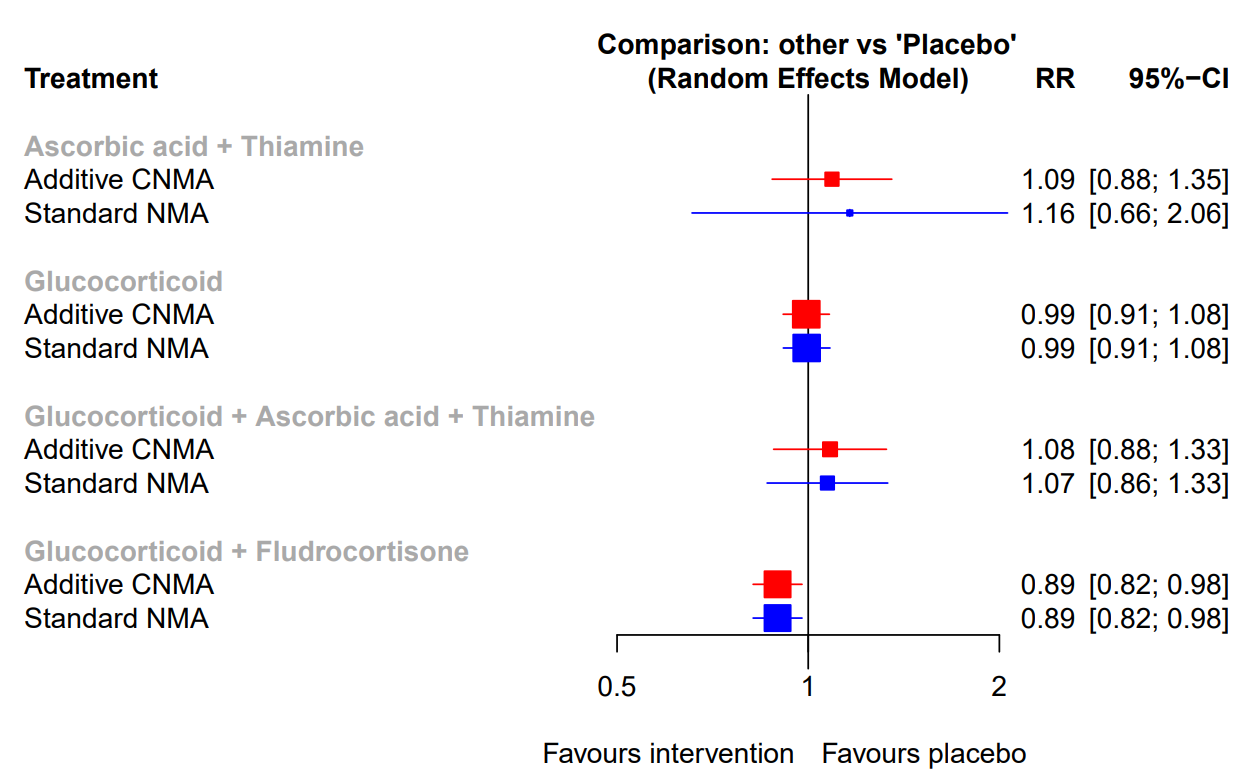


Figure 8. Forest plot for components on longer-term mortality (≥ 90 days)


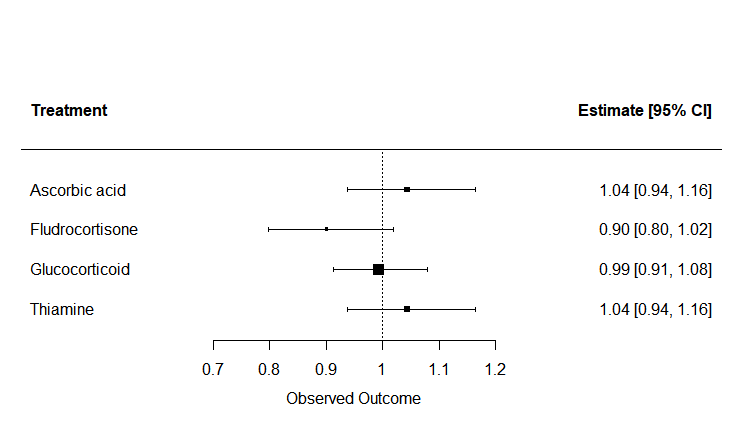


Estimate referred to risk ratio.

Figure 9. Network geometry of studies presenting time to resolution of shock


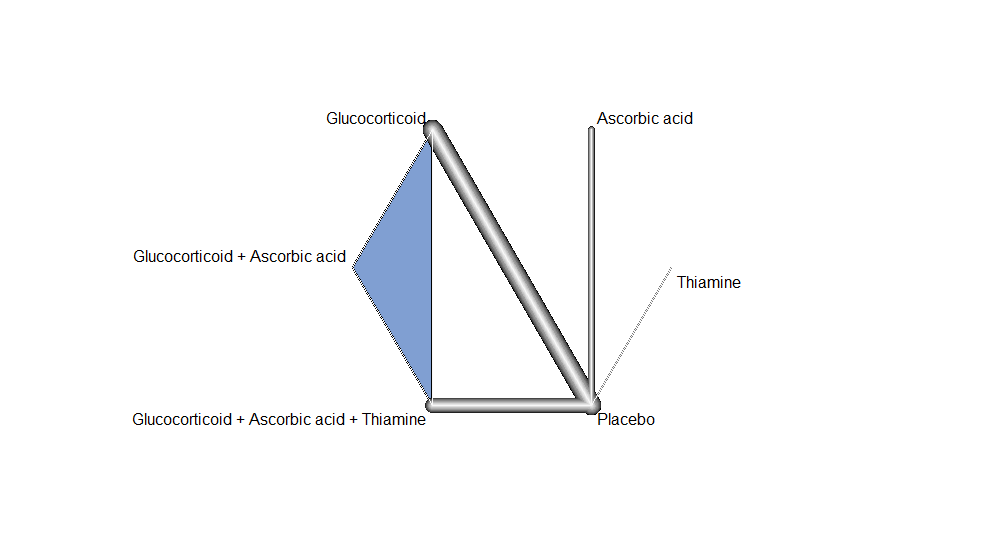


Figure 10. Forest plot for components in time to resolution of shock


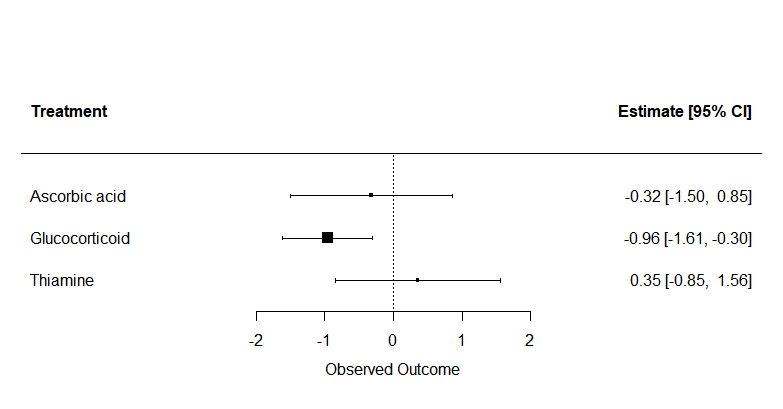
Estimate referred to mean difference.

Figure 11. Funnel plot on time to resolution of shock


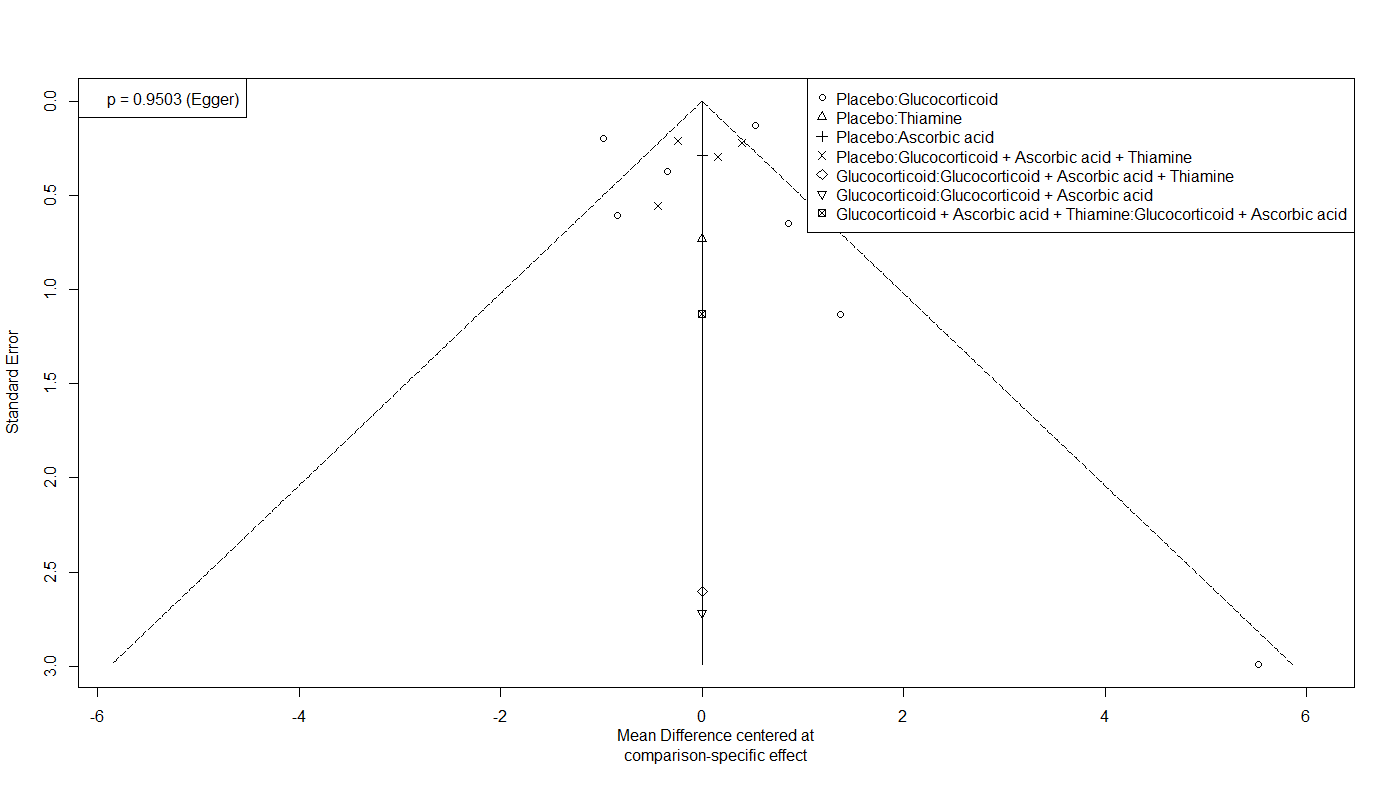


Figure 12. Network geometry on duration of mechanical ventilation


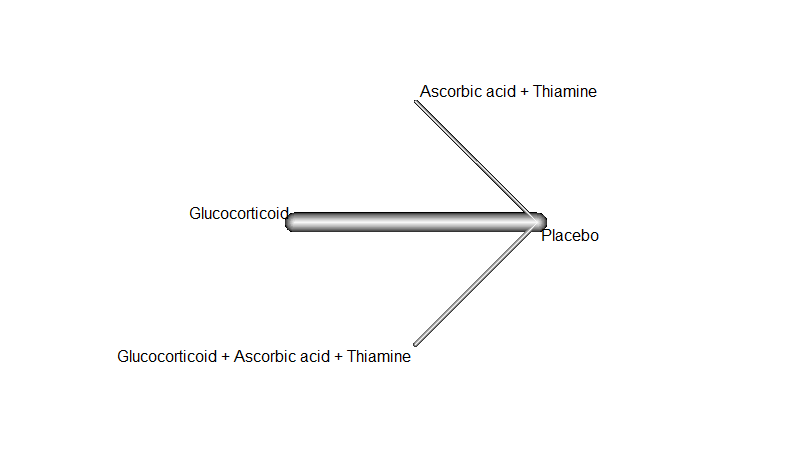


Figure 13. Network meta-analysis on duration of mechanical ventilation


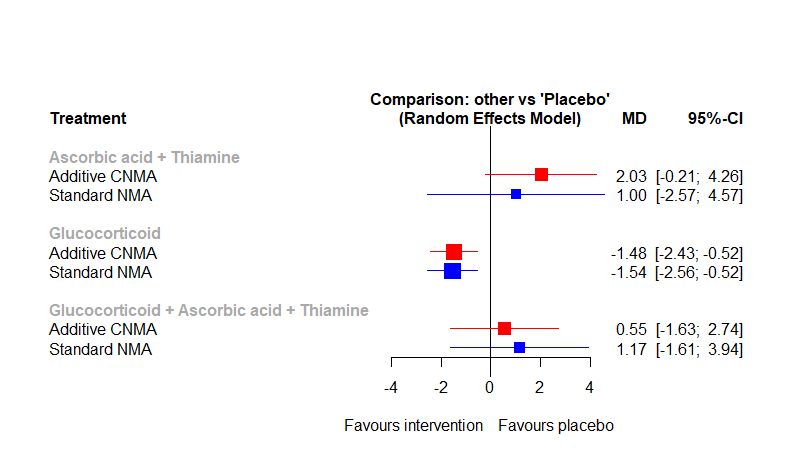


Figure 14. Forest plot for components on duration of mechanical ventilation


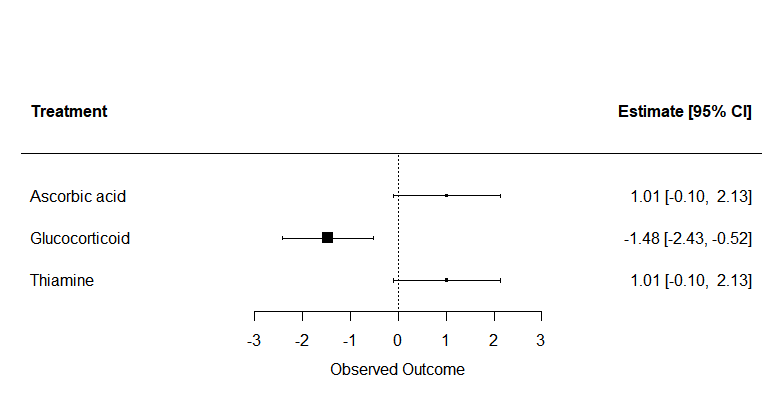


Estimates referred to mean difference.

Figure 15. Network geometry on ICU length of stay


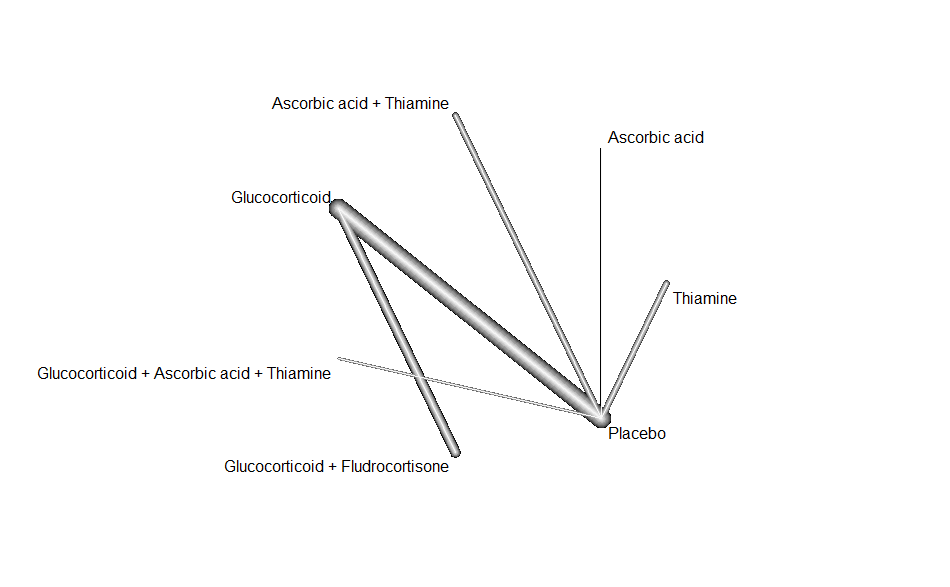


Figure 16. Network geometry on hospital length of stay


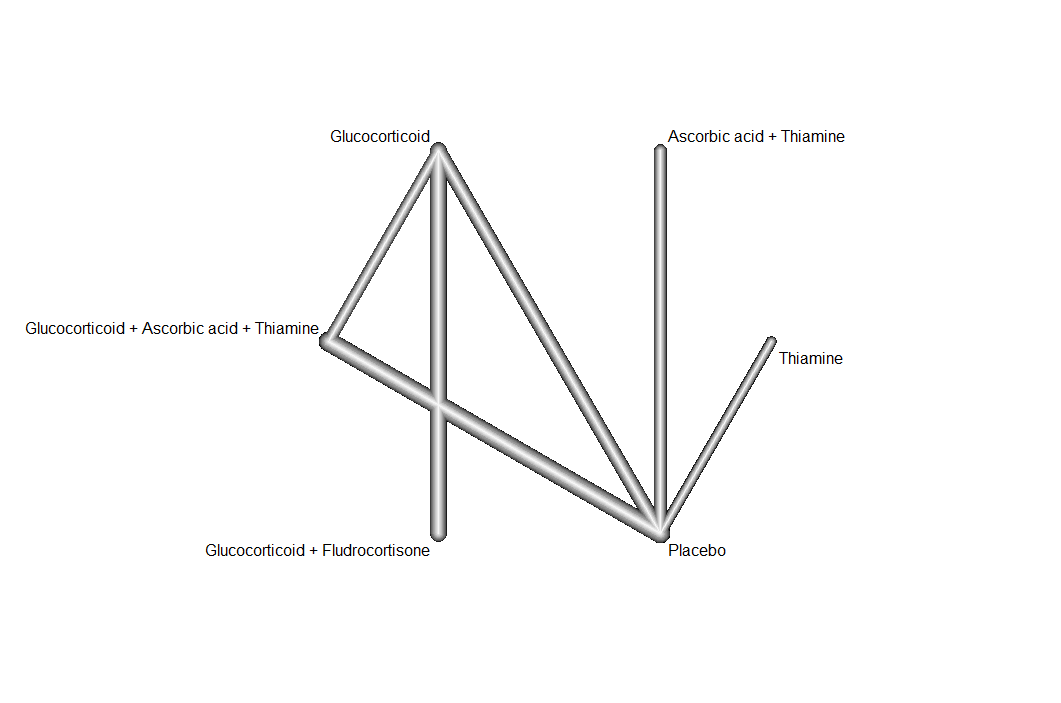


Figure 17. Network meta-analysis on ICU length of stay


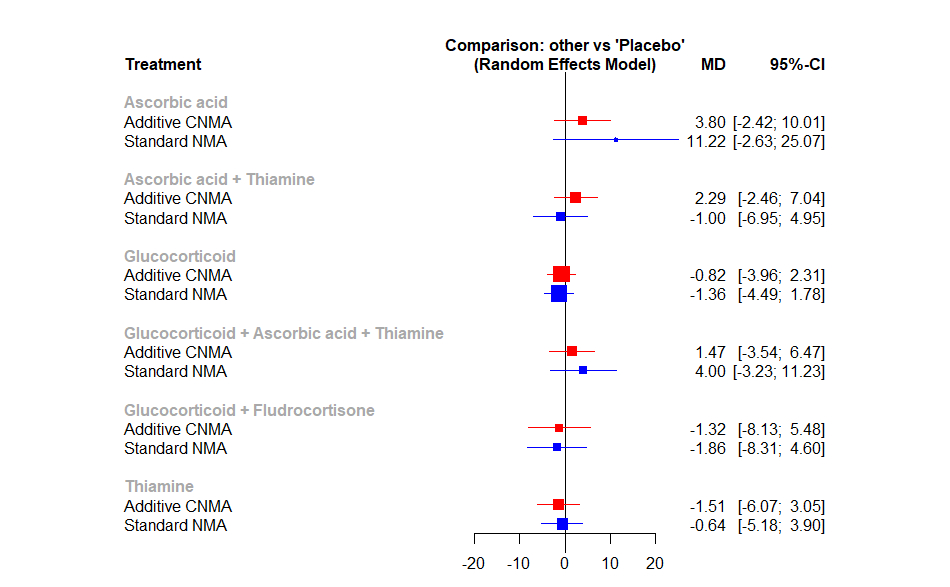


Figure 18. Network meta-analysis on hospital length of stay


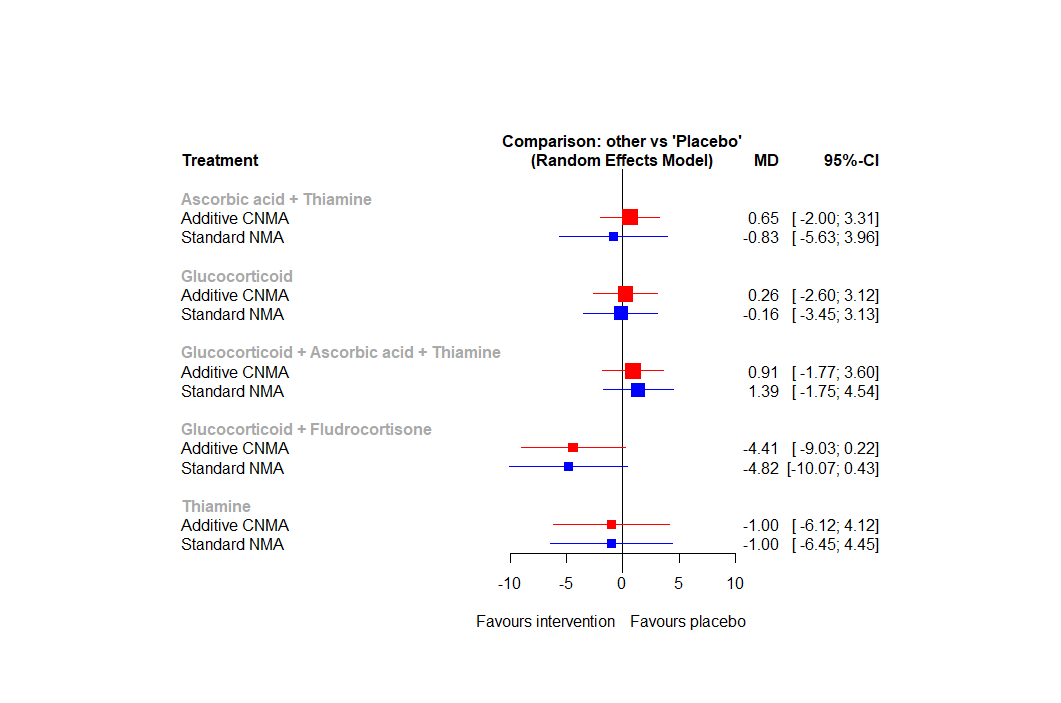


Figure 19. Funnel plot on ICU length of stay


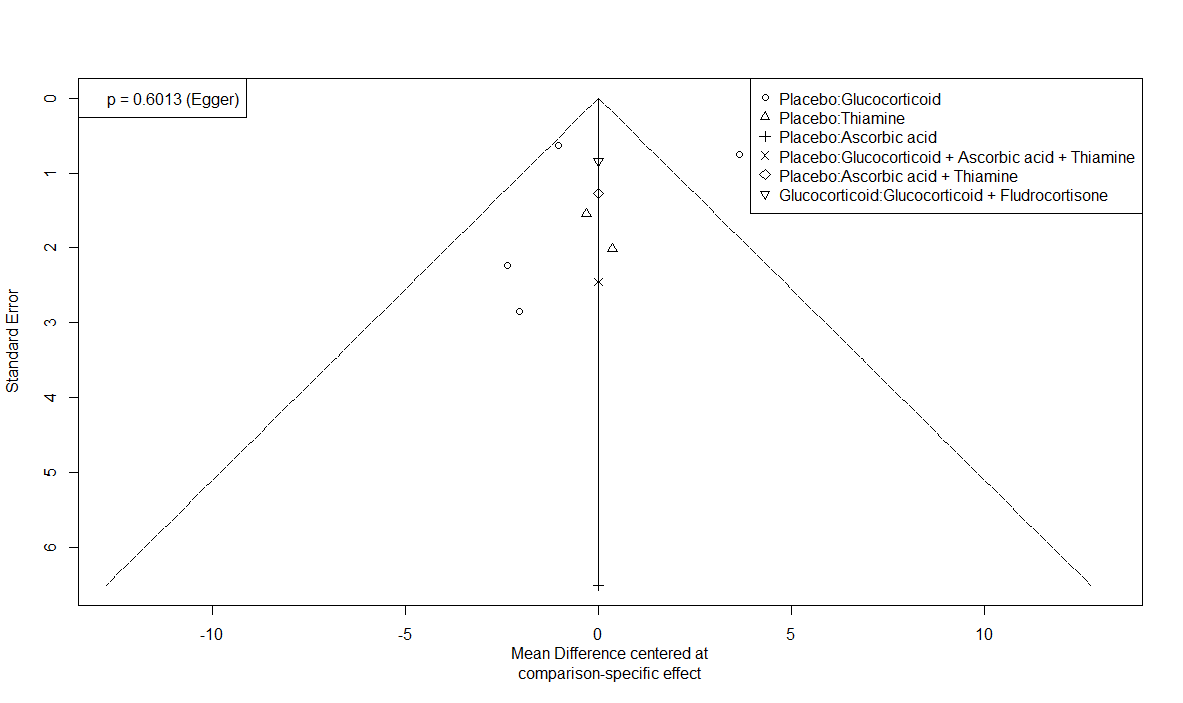


Figure 20. Adverse events related to steroid

(a)


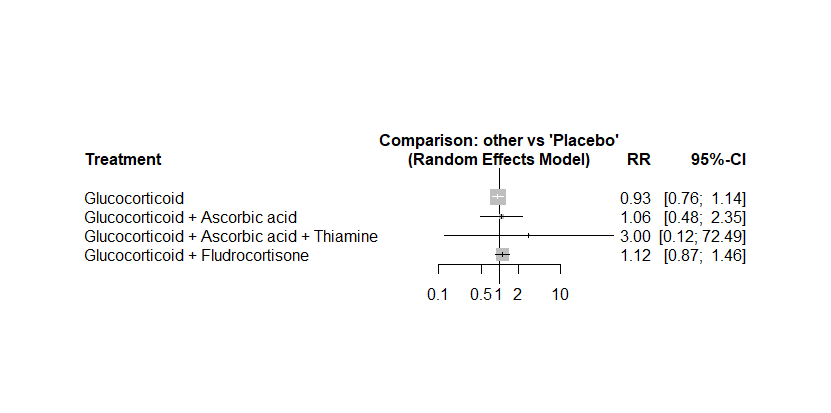


(b)


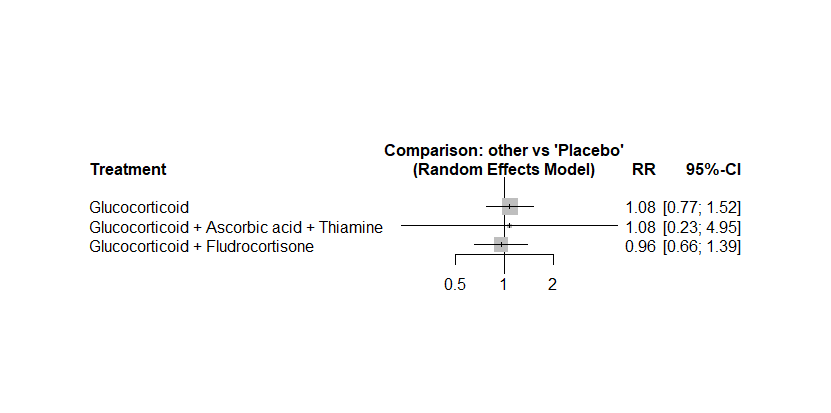


(c)


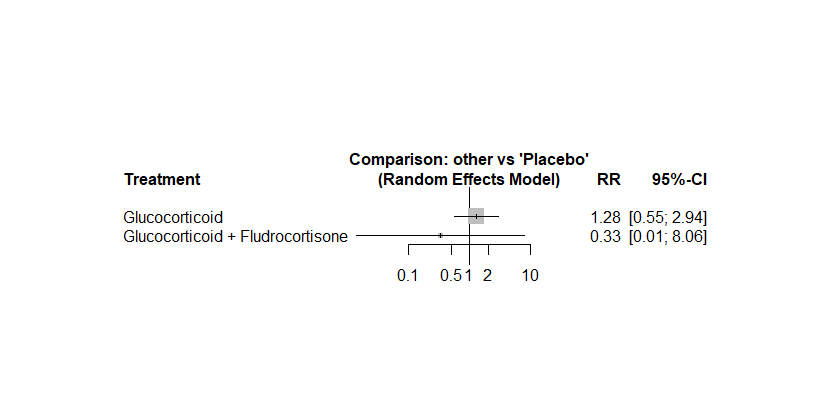


(d)


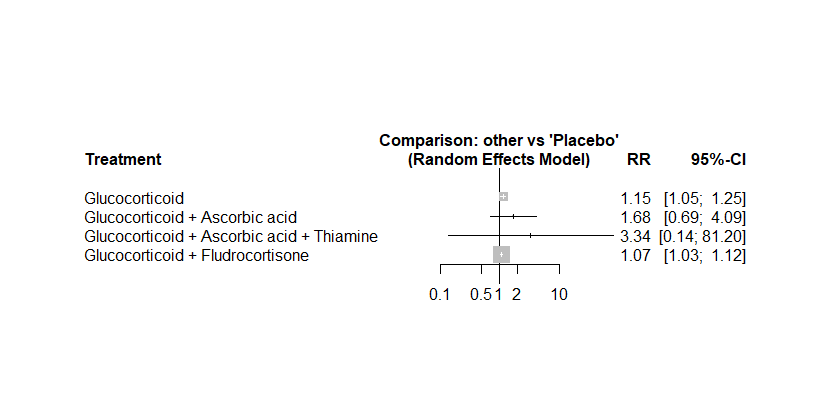


(e)


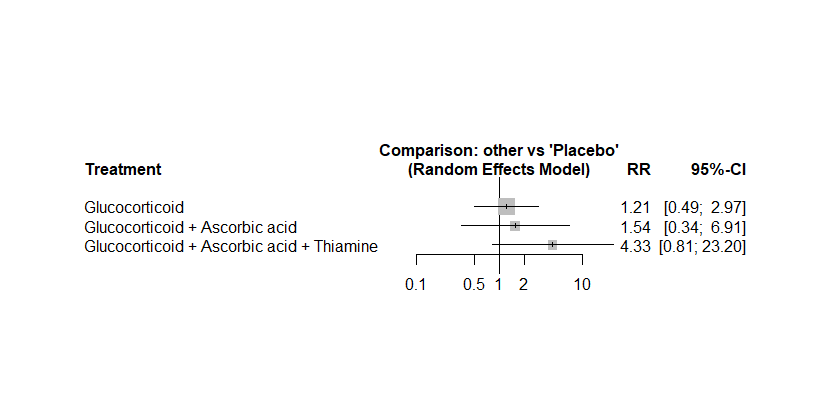


(a) Secondary infections, (b) Gastrointestinal bleeding, (c) Delirium, (d) Hyperglycemia, (e) Hypernatremia

Figure 21. Sensitivity analysis of network meta-analysis on short-term mortality limiting to studies of low risk of bias


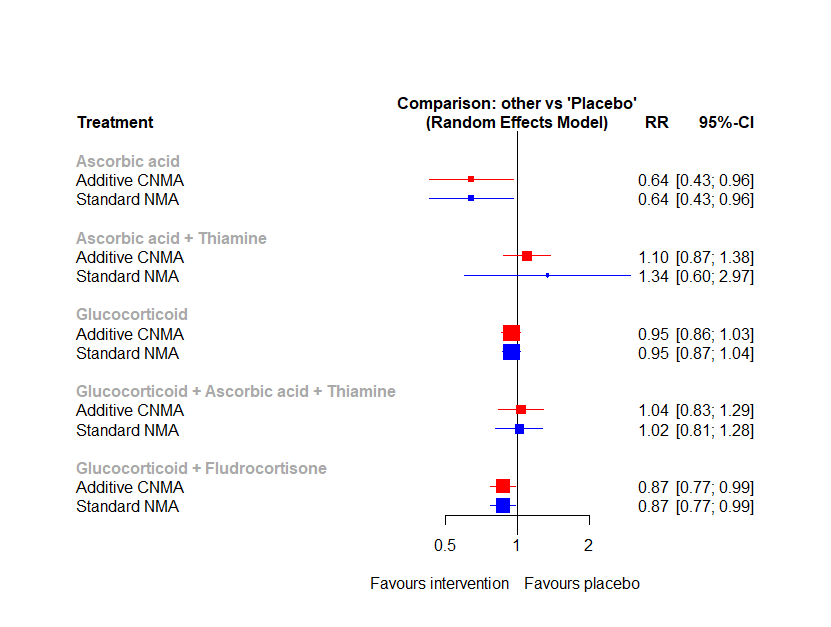


Figure 22. Sensitivity analysis of network meta-analysis on short-term mortality limiting to studies recruiting >50% of patients dependent on inotrope/ vasopressor


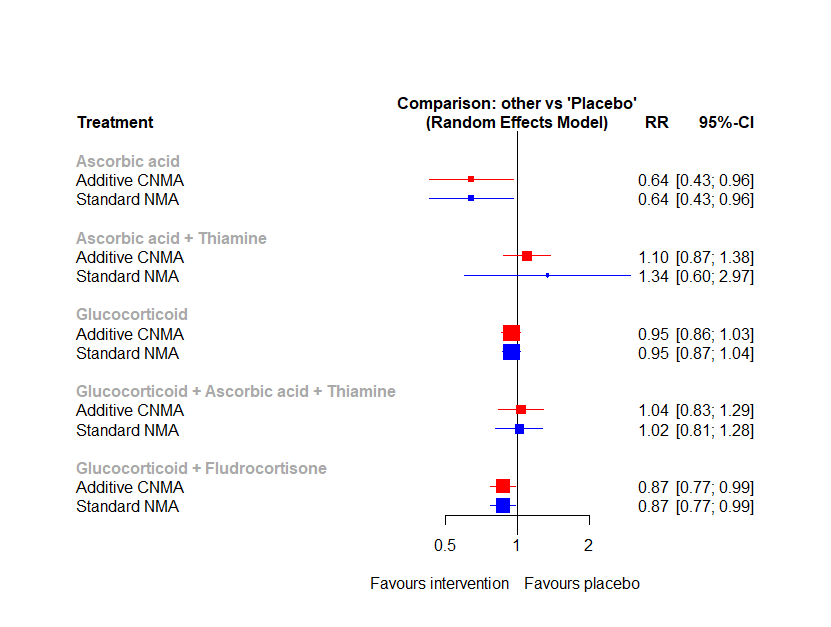


Figure 23. Sensitivity analysis of network meta-analysis on short-term mortality limiting to studies recruiting patients after 2016


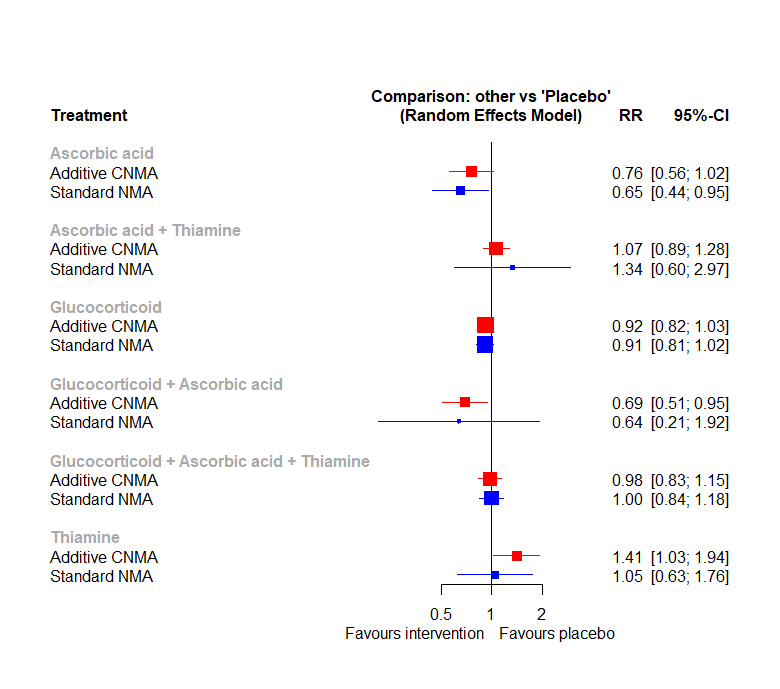


Figure 24 Sensitivity analysis of network meta-analysis on short-term mortality excluding studies using high dose corticosteroid (≥400mg/day hydrocortisone or equivalent)


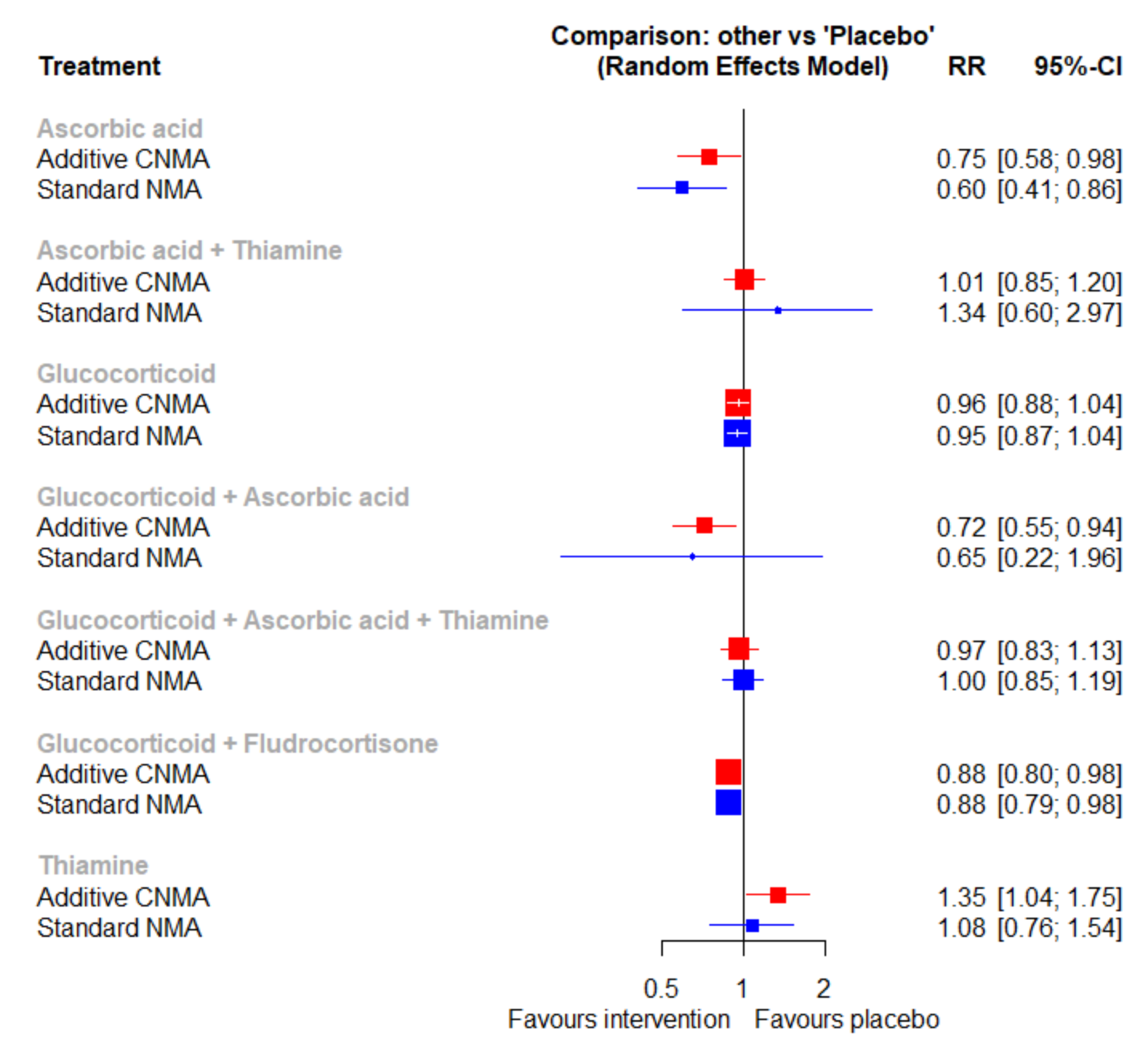


Figure 25 Sensitivity analysis of network meta-analysis on time to resolution of shock excluding studies using high dose corticosteroid (≥400mg/day hydrocortisone or equivalent)


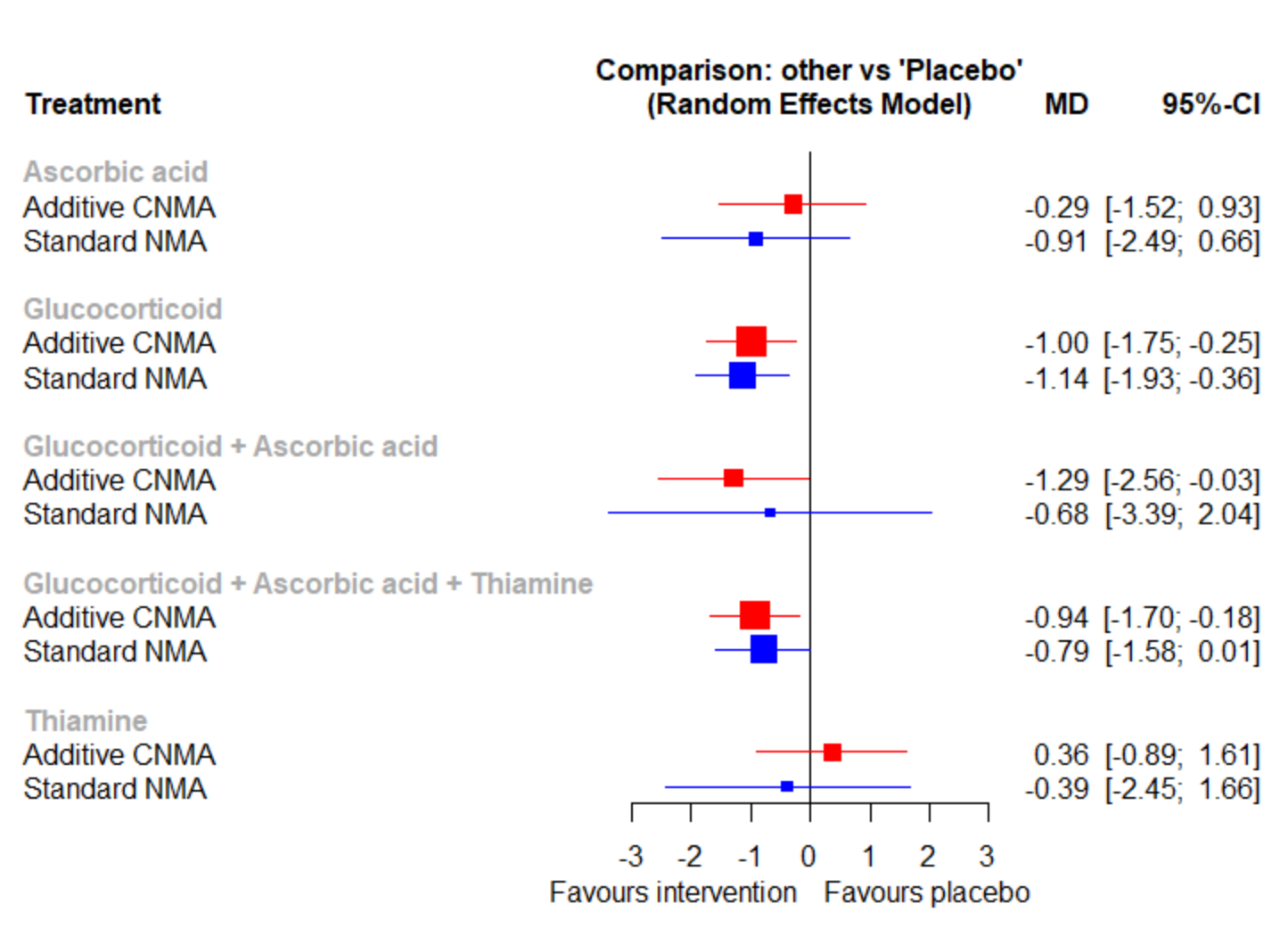


Figure 26 Sensitivity analysis of network meta-analysis on time of mechanical ventilation excluding studies using high dose corticosteroid (≥400mg/day hydrocortisone or equivalent)


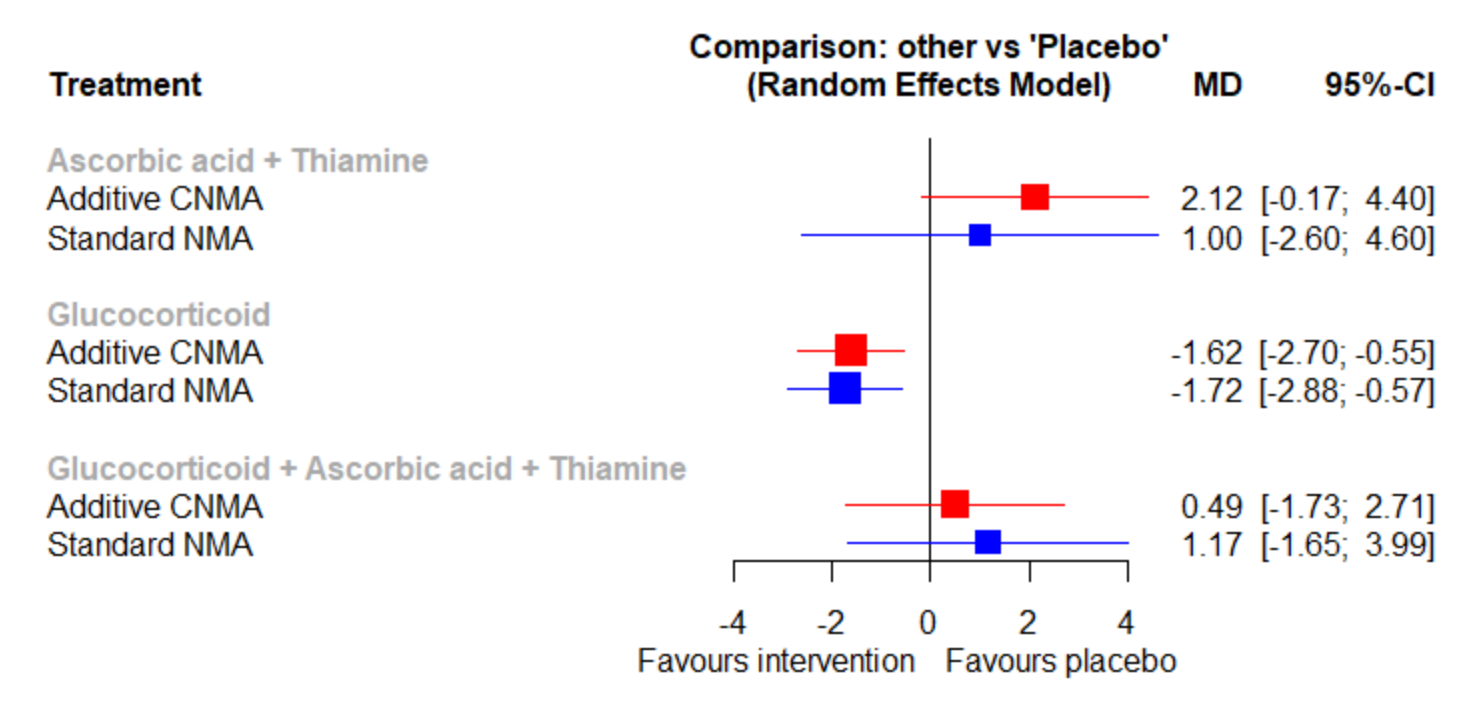


Reference

1. Higgins J, Li T, Deeks Je. Chapter 6: Choosing effect measures and computing estimates of effect. In: Higgins J, Thomas J, Chandler J, Cumpston M, Li T, Page M, et al., editors. Cochrane Handbook for Systematic Reviews of Interventions version 60 (updated July 2019)2019.

2. Wan X, Wang W, Liu J, Tong T. Estimating the sample mean and standard deviation from the sample size, median, range and/or interquartile range. BMC Med Res Methodol. 2014;14:135.
